# Effect of Cross-Link Homogeneity on the High-Strain Behavior of Elastic Polymer Networks

**DOI:** 10.1021/acs.macromol.3c02565

**Published:** 2024-05-08

**Authors:** Victoria A. Kong, Thomas A. Staunton, Jennifer E. Laaser

**Affiliations:** Department of Chemistry, University of Pittsburgh, Pittsburgh, Pennsylvania 15260, United States

## Abstract

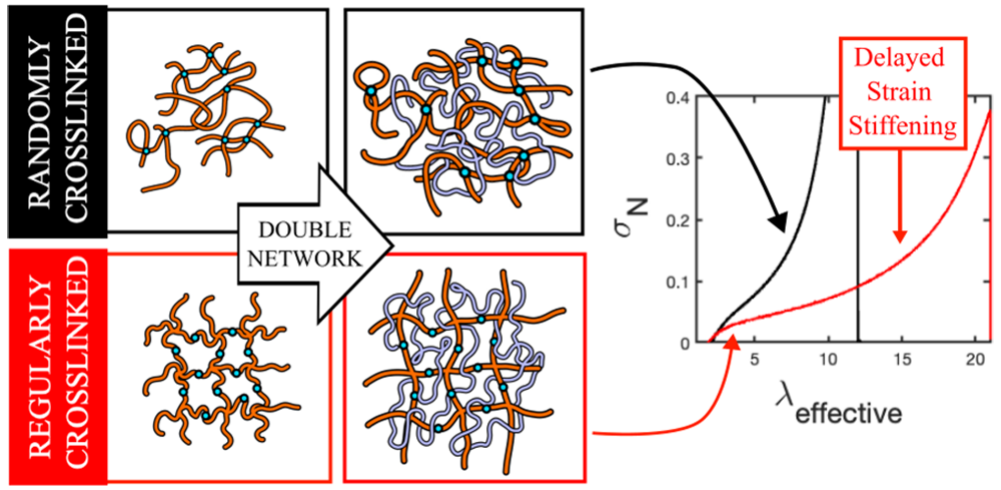

Cross-link heterogeneity and topological defects have been shown to affect the moduli of polymer networks in the low-strain regime. Probing their role in the high-strain regime, however, has been difficult because of premature network fracture. Here, we address this problem by using a double-network approach to investigate the high-strain behavior of both randomly and regularly cross-linked networks with the same backbone chemistry. Randomly cross-linked poly(*n*-butyl acrylate) networks with target molecular weights between cross-links of 5–30 kg/mol were synthesized via free-radical polymerization, while regularly cross-linked poly(*n*-butyl acrylate) networks with molecular weights between cross-links of 7–38 kg/mol were synthesized via cross-linking of tetrafunctional star polymers. Both types of networks were then swollen in a monomer/cross-linker mixture, polymerized to form double networks, and characterized via uniaxial tensile testing. The onset of strain stiffening was found to occur later in regular networks than in random networks with the same modulus but was well-predicted by the target molecular weight between cross-links of each sample. These results indicate that the low- and high-strain behavior of polymer networks result from different molecular-scale features of the material and suggest that controlling network architecture offers new opportunities to both further fundamental understanding of architecture–property relationships and design materials with independently controlled moduli and strain stiffening responses.

## Introduction

Polymer networks are used in a broad range of applications, including but not limited to soft electronics,^1−3^ shape memory materials,^4−6^ and tissue engineering scaffolds.^7−9^ For many of these applications, the polymer networks must withstand high strain deformations.^10,11^ While the high-strain responses of biological gels, which are often composed of stiff, fibrillar biopolymers, have received significant attention,^12−16^ the features of flexible polymer networks that determine their behavior in this limit are still not well understood.^17,18^ Developing a detailed picture of how network structure determines the mechanical properties of these materials at high strain is thus critical both for optimizing their use in high-performance applications and for advancing fundamental understanding of structure–property relationships in cross-linked polymeric materials.^19−22^

The majority of the literature on the mechanical properties of flexible polymer networks to date has focused on networks synthesized via cross-linking of natural rubbers or via free-radical or step growth polymerizations of small monomers and oligomers. These synthetic routes generally produce networks with highly heterogeneous cross-link densities and network strand lengths^23−25^ and allow control of the network properties only through broad handles such as the overall cross-link density (and, correspondingly, the average molecular weight between cross-links).^26,27^ In these systems, increasing the average molecular weight between cross-links typically decreases the modulus of the materials and increases their extensibility.^28−31^ Using randomly cross-linked networks as a platform for understanding the relationship between molecular-scale features of networks and their bulk properties, however, has a number of limitations. In addition to the average molecular weight between cross-links, a number of other features of the network, such as the presence of defects, distribution of network strand lengths, and spatial heterogeneity of the cross-link density, may play an important role in the mechanical properties of networks at both low and high strain, and these features are difficult if not impossible to control in typical random network syntheses.

Recently, a number of groups have shown that many of these variables can be controlled in networks synthesized by cross-linking monodisperse polymeric precursors via highly efficient “click” reactions such as azide–alkyne,^32−34^ activated ester–amine,^35−38^ and thiol–ene reactions^39,40^ under carefully controlled solution conditions. This approach yields regularly cross-linked networks with moduli significantly closer to the theoretical limits and serves as an excellent platform for elucidating the impact that features such as topology and chain length distribution have on the mechanics of the materials. For example, in 2016, Zhong et al. developed the Real Elastic Network Theory, which captures the effect of loop defects in elastic networks, and tested it in regularly cross-linked PEG networks using rheology, network disassembly spectroscopy, and simulations.^34^ They found that increasing the loop defect content of the networks decreased their moduli because of the decrease in the density of elastically effective strands.^41^ Computational models have also suggested that the dispersity of the network strands plays an important role. Tehrani et al. found, for example, that in a network with a broad dispersity of strand lengths, shorter chains quickly reached the limit of their extensibility and broke, even under small strains.^27^ This leads us to hypothesize that the strain stiffening behavior of regular and random networks should be different, even if they have the same modulus and the same apparent average molecular weight between cross-links. Randomly cross-linked networks contain a polydisperse distribution of strand lengths with a high fraction of short strands and thus should strain harden at relatively low extensions. Regular networks, on the other hand, contain a much narrower distribution of predominantly higher molecular weight strands and should exhibit significantly delayed strain stiffening relative to comparable random networks.

Testing this hypothesis is challenging for two reasons. First, the synthesis of random and regular networks with the same backbone chemistry has been challenging. Most regularly cross-linked networks reported to date have been prepared from poly(ethylene oxide) (PEO) precursors,^32,34−37,39,40^ but the polymerizations required to prepare PEO backbones do not lend themselves well to synthesis of equivalent randomly cross-linked networks. Baba et al. attempted to address this issue by comparing *n*-butyl acrylate networks prepared by free-radical polymerization of monomer and cross-linker to networks prepared by chain-extending *n*-butyl acrylate star polymers with diacrylate cross-linkers. They found that the star polymer method yielded networks with higher fracture energy, but interpretation of the results was complicated by reaction of multiple difunctional cross-linkers at each junction point, which led to the apparent formation of nanofiller-like structures in the material.^42^ Recently, however, Huang et al. showed that thiol-bromine reactions could also be used to directly cross-link tetrafunctional *n*-butyl acrylate star polymers, yielding uniform networks with moduli close to the phantom network model predictions.^43^ Second, quantifying the high-strain behavior of polymer networks is often difficult, because of premature network fracture. This issue can be overcome, however, by toughening the network of interest with a second, loosely cross-linked interpenetrating network (IPN), which serves as a secondary stress dissipation mechanism. In 2003, Gong et al. showed that when IPN hydrogels of poly(2-acrylamido-2-methylpropanesulfonic acid) and poly(acrylamide) are synthesized with a high cross-link density in the first network and a low cross-link density in the second network, the material exhibited significantly improved toughness compared to conventional IPN hydrogels.^44^ Ducrot et al. later applied this approach to multinetwork elastomers and found that embedding a brittle elastomeric network in a softer, more extensible network significantly reduced premature network fracture^45,46^ and enabled characterization of the high strain behavior of the brittle first networks.^47^

Here, we combine these two approaches to investigate the effect of cross-link homogeneity on the high-strain behavior of randomly and regularly cross-linked networks with the same backbone chemistry and test the hypothesis that regular networks should exhibit delayed strain stiffening relative to random networks with the same cross-link density. We prepare randomly cross-linked networks of poly(*n*-butyl acrylate) via thermally initiated free-radical polymerization and regularly cross-linked networks via thiol-bromine coupling of tetrafunctional poly(*n*-butyl acrylate) star polymers. We then swell both types of networks in a mixture of monomer and cross-linker, which is polymerized to form double networks. We characterize the resulting materials using uniaxial tensile tests and find that double networks prepared from randomly and regularly cross-linked networks with the same modulus have significantly different mechanical properties at high strain. In particular, double-network elastomers prepared from regular networks are significantly more extensible and exhibit a delayed onset of strain stiffening compared to those prepared from random networks. From a fundamental perspective, these results highlight the fact that the low- and high-strain behaviors of cross-linked networks arise from different molecular-scale features of the materials, and understanding how the network architecture affects both the density of elastically effective strands and the length of those strands is critical for understanding the stress–strain responses of networks across broad ranges of deformations. From an application perspective, these results also provide insight into how network topology can be used to independently tune both the low- and high-strain responses of polymeric materials, broadening the design possibilities for networks used in demanding, high-performance applications.

## Experimental Methods

### Synthesis

**Scheme 1:**
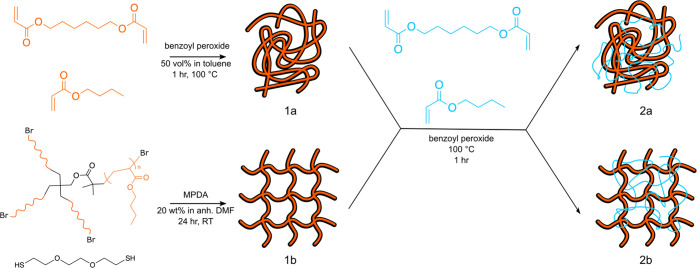
General Approach to the Synthesis of (1a, 2a) Randomly and (1b, 2b) Regularly Cross-Linked (1a, 1b) Single Networks and (2a, 2b) Double Networks

The synthetic approach used in this work is summarized in Scheme 1. Briefly, randomly cross-linked networks were synthesized by free-radical polymerization of *n*-butyl acrylate and hexanediol diacrylate. Regularly cross-linked networks were synthesized by cross-linking tetrafunctional poly(*n*-butyl acrylate) star polymers. Both network types were then swollen in a monomer/cross-linker mixture and polymerized to prepare the targeted double networks, as described in more detail, below.

### Single Networks

#### Randomly Cross-Linked Networks

Randomly cross-linked networks were synthesized by free radical polymerization of *n*-butyl acrylate (monomer), hexanediol diacrylate (cross-linker), and benzoyl peroxide (initiator) in toluene. The target molecular weight between cross-links, *M*_*x*_, of each network was set by varying the amount of cross-linker added to the reaction mixture (see Supporting Information). The reagents were degassed, combined under an inert atmosphere, injected into a sealed mold, and placed in a 100 °C oven for 1 h to polymerize. The samples were then removed from the oven, released from the mold, and soaked in a solvent bath to remove any unreacted monomer. The samples were finally placed in a warm oven to dry fully before use. Detailed reaction conditions for synthesis of the randomly cross-linked networks are provided in the Supporting Information.

#### Tetrafunctional Star Polymers

Bromine-functionalized poly(*n*-butyl acrylate) star polymers were synthesized using a single-electron transfer living radical polymerization (SET-LRP).^48,49^ Briefly, inhibitor-free *n*-butyl acrylate (monomer), tris[2-(dimethylamino)ethyl]amine (ligand), pentaerythritol tetrakis(2-bromoisobutyrate) (tetrafunctional initiator), CuBr_2_ (deactivator), and a stir bar wrapped in activated copper wire (catalyst) were combined in anhydrous dimethyl sulfoxide and allowed to stir for 24 h at room temperature. The crude polymer was precipitated into a cold ethanol and water mix and run through a silica column to remove residual CuBr_2_. The polymer was then thoroughly dried under vacuum before use. Detailed reaction conditions for synthesis of the tetrafunctional star polymers are provided in the Supporting Information.

#### Regularly Cross-Linked Networks

Regularly cross-linked networks were synthesized using a thiol-bromine click reaction between the bromine-terminated tetrafunctional poly(*n*-butyl acrylate) star polymers and 3,6-dioxa-1,8-octanedithiol cross-linker in the presence of *N*-methyl-1,3-diaminopropane (MPDA) base. Briefly, a stoichiometrically equivalent amount (relative to reactive groups) of polymer and dithiol were dissolved in anhydrous dimethylformamide (DMF) in a glovebox at a concentration of 20 wt % (approximately 2–4× the overlap concentration of the star polymers; see Supporting Information), then combined with an aliquot of MPDA diluted in additional anhydrous DMF. The resulting solutions were poured into Teflon molds and allowed to react for 24 h in the glovebox. The gelled samples were then removed from the molds and soaked in an acetone/methanol bath to remove DMF and MPDA. The samples were then dried in a warm vacuum oven before use. Detailed reaction conditions for synthesis of the regularly cross-linked networks are provided in the Supporting Information.

### Double Networks

Double-network samples were prepared by free-radical polymerization of a monomer/cross-linker mixture used to swell the random or regularly cross-linked network of interest, as described by Ducrot et al.^47,50^ Briefly, inhibitor-free *n*-butyl acrylate (monomer), hexanediol diacrylate (cross-linker), and benzoyl peroxide (initiator) were combined in a ratio of 10000:1:1. The random or regularly cross-linked network of interest was fully submerged in this solution and allowed to swell to equilibrium. Samples were then removed from the bath, blotted dry, and sealed between two PTFE sheets clamped to glass plates. The samples were placed in a 100 °C oven for 1 h to initiate polymerization of the second network. Samples were then removed from the oven, released from the glass plates, and held at 100 °C overnight to evaporate off unreacted monomer and cross-linker. Samples were finally placed under vacuum for an additional 6–8 h to remove any remaining monomer before use. Detailed reaction conditions for synthesis of the double networks are provided in the Supporting Information.

### Characterization

#### Mechanical Tests

Networks were cut into dog-bone-shaped tensile samples and analyzed using a uniaxial extension test (1 in/min) on either an ADMET eXpert 5601-2C or a Material Testing Systems Insight Dual Transformer 820 XFMR-DUAL mechanical tester. The modulus of each sample was calculated using a linear fit to the stress–strain data at extension ratios below 1.2 and averaged over 3–5 samples.

## Results

### Single Networks

#### Randomly Cross-Linked

Randomly cross-linked networks with target molecular weights between cross-links (*M*_*x*_) of 5–30 kg/mol were synthesized using thermally initiated free-radical polymerization. The monomer (*n*-butyl acrylate) and initiator (benzoyl peroxide) concentrations were held constant for all samples, and *M*_*x*_ was controlled by varying the amount of cross-linker (hexanediol diacrylate) added to the mixture (see Supporting Information). The resulting samples and their targeted molecular weights between cross-links are summarized in Table 1. In this table, and throughout the text, these samples are denoted RAN_*x*_, where RAN indicates the cross-link type (random) and the subscript indicates the targeted *M*_*x*_ in kg/mol (ex: *RAN*_5_ is a randomly cross-linked network with a targeted molecular weight between cross-links of 5 kg/mol).

**Table 1:** Theoretical and Experimental Moduli of Randomly Cross-Linked Single Networks

	theoretical		experimental	
sample	*M*_*x*_ [kg/mol]	*E* [kPa]	*E* [kPa]	% of theoretical *E*
RAN_5_	5	946	148 ± 9	15 ± 1
RAN_8.8_	8.8	538	87 ± 6	16 ± 1
RAN_10_	10	473	63 ± 3	13 ± 1
RAN_15_	15	315	49 ± 3	16 ± 1
RAN_20_	20	237	33 ± 5	14 ± 2
RAN_25_	25	189	35 ± 4	18 ± 2
RAN_30_	30	158	27 ± 8	17 ± 5

**Figure 1:**
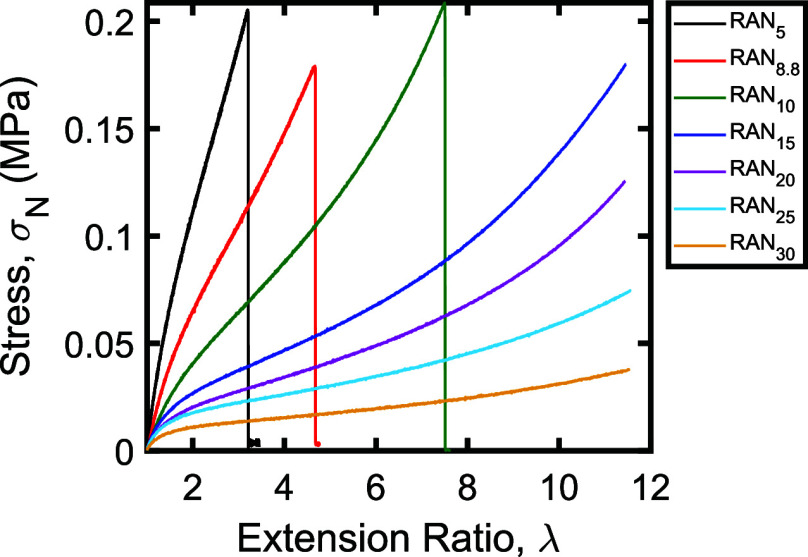
Stress–strain curves of randomly cross-linked single networks. Samples were extended either until network fracture (RAN_5_–RAN_10_) or until the instrument reached its maximum extension near λ = 12 (RAN_15_–RAN_30_). Plotted data were smoothed using a Fourier filter to remove instrument noise and minimize overlap between the traces (see Supporting Information).

Stress–strain curves for the resulting networks are shown in Figure 1. As seen in this figure, three distinct regimes are observed in the stress–strain curves of the samples: a linear elastic regime at low strain, a plateau at intermediate strain, and then a sharp increase in force at high strain, as the samples undergo strain stiffening. This strain stiffening regime is most obvious in the RAN_10_, RAN_15_, and RAN_20_ samples. While strain stiffening is expected in all of the networks, samples with a higher targeted *M*_*x*_ (such as RAN_25_ and RAN_30_) reached the limit of the extension for the mechanical tester before a clear onset of strain stiffening was observed. Low *M*_*x*_ samples such as RAN_5_ and RAN_8.8_ fractured too early to exhibit noticeable strain stiffening during the tensile test. As expected, the strain at break, or overall extensibility of the networks, also increased as the targeted *M*_*x*_ increased.

The portions of the stress–strain curves below λ = 1.2 were then fit to determine the Young’s moduli of the materials, as summarized in Table 1. The measured moduli were compared to the expected moduli, calculated using
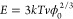
1where
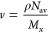
2is the number density of elastically effective strands per unit volume in a dry network with average molecular weight between cross-links *M*_*x*_ and density ρ (here, ρ = 1080 kg/m^3^ for bulk poly(*n*-butyl acrylate)), and ϕ_0_ is the volume fraction of polymer at which the network was prepared. We note that inclusion of the ϕ_0_^2/3^ term is necessary to account for the fact that the networks were synthesized in solvent and deswollen prior to characterization (see Supporting Information); for the RAN networks reported here, which were synthesized at 50 vol % monomer in toluene, ϕ_0_ ≈ 0.453. Consistent with prior work and the measured gel fractions of the samples (see Supporting Information),^34^ we find that the experimental moduli of the randomly cross-linked networks are substantially lower than the expected moduli, with the moduli of the networks reaching only 13–18% of their theoretical values. We note, however, that strain rate effects and contributions from entanglements in the samples with *M*_*x*_ > 15 kg/mol (see Supporting Information) may mean that the part of the modulus attributable to covalent cross-links is even lower than these numbers suggest.

#### Regularly Cross-Linked

Regularly cross-linked single networks were synthesized by cross-linking tetrafunctional poly(*n*-butyl acrylate) star polymers. The polymers had molecular weights ranging from 14 to 77 kg/mol, giving rise to networks with expected molecular weights between cross-links of 7–38 kg/mol (note: each cross-link strand is formed from two arms on different star polymers, so *M*_*x*_ is  of the four-arm star polymer). The properties of the star polymers and the resulting networks are summarized in Table 2. As with the randomly cross-linked networks, the regularly cross-linked networks are labeled REG_*x*_, where REG indicates the cross-link type and the subscript indicates the expected *M*_*x*_ of the sample in kg/mol.

**Table 2:** Properties of Tetrafunctional Poly(*n*-butyl acrylate) Star Polymer Precursors and Resulting Regularly Cross-Linked Single Networks

	precursor polymer			theoretical		experimental	
sample	*M*_n_ [kg/mol]	*M*_w_ [kg/mol]	Đ	*M*_*x*_ [kg/mol]	*E* [kPa]	*E* [kPa]	% of theoretical modulus
REG_7_	14^a^	15^a^	1.03^a^	7	365	143 ± 23	39 ± 6
REG_20_	40^a^	41^a^	1.03^a^	20	128	105 ± 4	82 ± 3
REG_26_	52^b^	59^b^	1.13^b^	26	98	66 ± 3	67 ± 3
REG_38_	77^b^	89^b^	1.16^b^	38	66	57 ± 6	86 ± 9

a Analyzed by size exclusion chromatography (TOSOH ECO-SEC; eluent: THF; temperature: 40 °C; absolute molecular weight analysis using Wyatt Dawn8+ light scattering detector with d*n*/d*c* = 0.067).

b Analyzed by MALDI-TOF (matrix: IAA/Na^+^TFA); method: LP5-50kDa)

**Figure 2:**
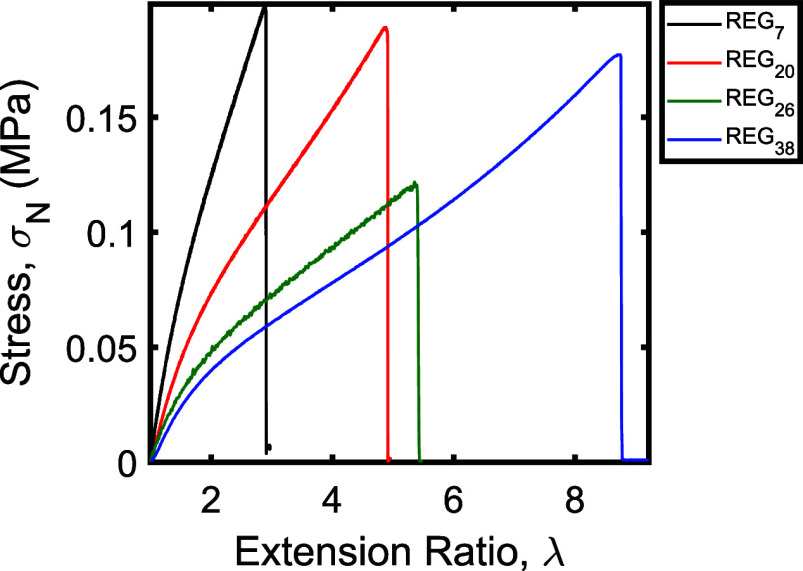
Stress–strain curves of regularly cross-linked single networks. All samples were extended until network fracture. Plotted data were smoothed using a Fourier filter to remove instrument noise and minimize overlap between the traces (see Supporting Information).

Stress–strain curves for these networks are shown in Figure 2. As seen in this data, the tensile responses were generally similar to those observed for the randomly cross-linked networks with low target molecular weights between cross-links. As with the random networks, the strain at break increased with increasing target *M*_*x*_. Interestingly, however, in stark contrast to the randomly cross-linked networks with similar target *M*_*x*_, no significant sign of strain stiffening was observed prior to break in any of the regularly cross-linked samples. The regularly cross-linked networks also lacked the plateau in the stress–strain curves observed in many of the randomly cross-linked networks at intermediate strains; Mooney–Rivlin plots correspondingly indicate that entanglements played only a minor role in the mechanical responses of the regularly cross-linked samples (see Supporting Information).

As for the random networks, the low-strain data from the REG networks were fit to obtain the Young’s modulus of each material. The resulting moduli are summarized in Table 2, along with the theoretical moduli calculated from the molecular weight between cross-links and ϕ_0_ = 0.179 (corresponding to cross-linking at 20 wt % polymer in DMF). For the network with the lowest expected molecular weight between cross-links, REG_7_, the measured modulus was only 39% of the theoretical modulus. The samples with the higher molecular weights between cross-links (REG_20_, REG_25_, and REG_38_), on the other hand, reached between 67% and 86% of their theoretical moduli, significantly higher than values observed for the random networks and on par with previously reported regularly cross-linked star polymer networks.^37,43^

### Double Networks

**Figure 3:**
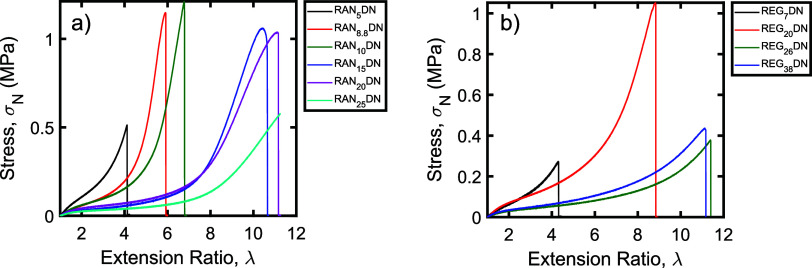
Stress–strain curves of double networks prepared from (a) randomly cross-linked and (b) regularly cross-linked first networks.

Duplicate samples of each of the networks characterized in the preceding sections were next swollen in a monomer/cross-linker mixture and polymerized to form double networks. We refer to the double networks prepared from each single-network sample as either RAN_*x*_DN or REG_*x*_DN, where the RAN or REG label and the subscript denote the first network from which the sample was prepared, and DN is appended to indicate that the label refers to the resulting double network. The stress–strain curves of the double networks are shown in Figure 3. As seen in these data, the stress–strain curves for the double networks generally exhibited the same three response regimes observed in the single-network samples. Importantly, however, strain stiffening behavior was observed in all of the DN samples, indicating that the double-network approach was indeed successful in delaying fracture enough to access the high-strain behavior of the underlying first network. We note that RAN_30_DN was excluded from the double-network analysis because it was too fragile for our uniaxial extension test setup.

To facilitate direct comparison of the single- and double-network data, and of double network data for samples with slightly different swelling ratios, the extension ratios from the double-network experiments were corrected to reflect the extension of the underlying first network alone, as described by Ducrot et al.^47,50^ The weight fraction of the first network (ϕ_*SN*_) within each double-network sample was first calculated using
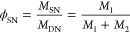
3where *M*_SN_ is the mass of the single network used to prepare the double network and *M*_DN_ is the total mass of the double-network sample. The swelling ratio of the first network, λ*, was then calculated using
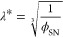
4where the assumption is made that the first network is isotropically swollen during the double-network synthesis. This swelling ratio reflects the degree of prestraining of the first network within the double-network sample; as shown in Table 3, all networks had swelling ratios close to 2. Finally, the effective extension ratio of the first network (λ_eff_) of each sample was calculated using
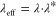
5This extension ratio reflects the total extension of the first network, taking into account both its degree of swelling in the as-synthesized double network and its deformation during the double-network tensile experiment.

**Table 3:** Randomly and Regularly Cross-Linked Double-Network Properties

sample	weight fraction (ϕ_SN_)	swelling ratio (λ*)
RAN_5_DN	0.18	1.77
RAN_8.8_DN	0.12	2.03
RAN_10_DN	0.14	1.93
RAN_15_DN	0.12	2.03
RAN_20_DN	0.12	2.03
RAN_25_DN	0.11	2.09
REG_7_DN	0.10	2.15
REG_20_DN	0.17	1.81
REG_26_DN	0.10	2.15
REG_38_DN	0.12	2.03

**Figure 4:**
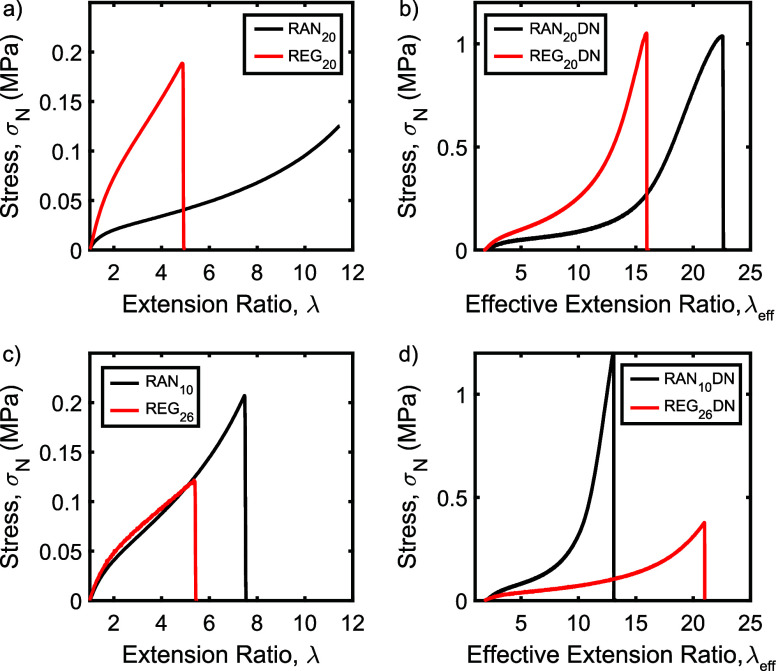
Representative stress–strain curves of (a, c) single and (b, d) double networks with matched (a, b) target molecular weight between cross-links and (c, d) modulus. Single-network data plotted in (a) and (c) were smoothed using a Fourier filter to remove instrument noise and minimize overlap between the traces (see Supporting Information).

The swelling-corrected double-network data were then used to directly compare the tensile responses of random and regular networks with either the same target molecular weights between cross-links or the same moduli. As shown in Figure 4(a), the REG_20_ single network was significantly stiffer than the RAN_20_ single network, as expected from the moduli reported in Tables 1 and 2; the REG_20_ network was also somewhat less extensible than the RAN_20_ network, consistent with trends predicted by coarse-grain simulations.^51^ Both of these trends observed in the single networks also translated to the behavior of the corresponding double-network samples, as shown in Figure 4(b), where the REG_20_DN network was also observed to strain-stiffen somewhat earlier than the RAN_20_DN network. By contrast, regular and random networks with the same modulus exhibited very similar behavior in their single networks but markedly different behavior in their double networks. The RAN_10_ and REG_26_ single-network samples had nearly identical moduli (*E* = 63 and 66 kPa, respectively), and their stress–strain curves (Figure 4(c)) were also comparable, exhibiting only slight shape differences above λ ≈ 1.5. Both single networks also fractured at similar extension ratios (λ ≈ 5.5–7.5). In the double-network samples (Figure 4(d)), however, significant differences emerged. The RAN_10_DN sample exhibited a pronounced strain stiffening response near λ_eff_ ≈ 10 and broke before λ_eff_ ≈ 14, while the REG_26_DN sample began to exhibit strain stiffening only around λ_eff_ ≈ 17, and the sample did not break until λ_eff_ ≈ 21.

To enable quantification of differences in the strain stiffening behavior of the random and regularly cross-linked network samples across the entire range of cross-link densities and moduli investigated in this work, the double-network data were fit to a swelling-modified version of the eight-chain model (see Supporting Information), which is a simple model for rubber elasticity in which the network is modeled using eight non-Gaussian chains along the diagonals of a rectangular coordinate system.^52^ In this analysis, the stress–strain curves for the randomly and regularly cross-linked double-network samples were fit to
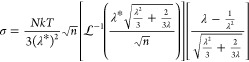
6where *N* is the number of elastically effective strands per unit volume, *n* is their degree of polymerization (in Kuhn monomers), *k* is the Boltzmann constant, *T* is the temperature,  is the inverse Langevin function, and λ* is the swelling ratio defined previously. Qualitatively, *NkT* determines the modulus and low-strain behavior of the material, while *n* determines its strain stiffening behavior. We note that direct interpretation of this model in terms of chain lengths in the double-network elastomers is challenging, as the shape of the strain stiffening response of these materials reflects the evolution of the network structure as bonds in the first network begin to break.^53^ As such, we use the fitted *n* values as a proxy for the onset of strain stiffening but caution that they cannot be rigorously interpreted as the actual degree of polymerization of the strands in the network. As shown in the representative fits in the Supporting Information, however, this model successfully fits the experimental data up to the onset of strain stiffening, suggesting that the *n* values extracted from the fits accurately capture the key differences in the strain stiffening behavior of the samples.

**Figure 5:**
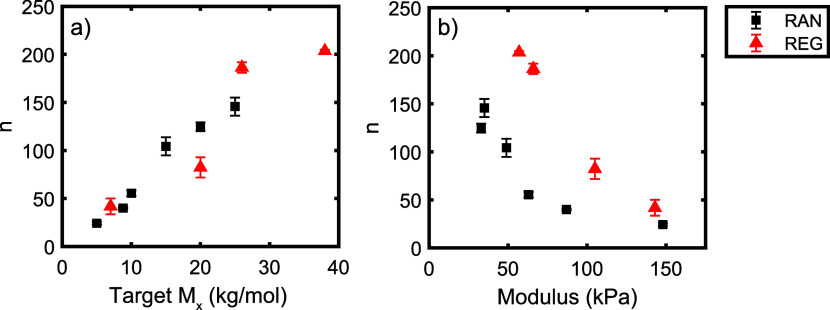
Fitted values of *n* as a function of (a) targeted *M*_*x*_ and (b) measured modulus for all double-network samples. Error bars represent the standard deviation of fits to data from multiple sample replicates (Supporting Information). Note that *n* is the fitted degree of polymerization in units of Kuhn monomers; plots with the *y* axis given in terms of equivalent molecular weight are included in the Supporting Information.

The fitted values of *n* for all double-network samples are summarized in Figure 5. As seen in this figure, fits to the regular network data yielded values of *n* approximately 2× larger than those obtained for random network samples with the same modulus. This result is consistent with the behavior shown in Figure 4(d), in which the regularly cross-linked sample (REG_26_DN) strain-stiffened later (and thus had a higher value of *n*) than the randomly cross-linked network (RAN_10_DN) with the same modulus. We note that this difference is not well-explained by the different polymer concentrations at which the RAN and REG first networks were synthesized (see Supporting Information) and must reflect the differences in the network topologies and/or strand length distributions of the RAN and REG samples. Interestingly, however, we note that the fitted *n* values were similar for regular and random networks with the same targeted molecular weight between cross-links, even when these networks had significantly different moduli. This result suggests the differences between the random and regular networks with the same targeted molecular weight between cross-links arise primarily from differences in their topology and defect content rather than from differences in the average strand length, as discussed in more detail, below. We note that while we are primarily interested in the strain stiffening behavior, as reflected by the fitted values of *n*, the fitted values of *NkT* were strongly correlated to the moduli of the materials across both network types (see Supporting Information), as expected.

## Discussion

In this work, we investigated the effect of cross-link homogeneity on the high-strain behavior of randomly and regularly cross-linked polymer networks. While increased cross-link homogeneity is known to improve the moduli of polymeric networks at low strains, its effect on the high-strain properties of these materials is not well-understood. Here, we addressed this problem by preparing regularly and randomly cross-linked networks of poly(*n*-butyl acrylate) and used a double-network strategy to access the strain stiffening regime. Critically, both the random and regular networks had the same backbone chemistry, enabling a direct comparison of their mechanical properties. We hypothesized that the double networks prepared from randomly cross-linked networks would strain-stiffen earlier than those prepared from regularly cross-linked networks with the same average molecular weight between cross-links, because the randomly cross-linked networks contain a higher fraction of short strands that should reach their maximum extensibility first. Interestingly, while we found that the double networks prepared from regularly cross-linked networks did indeed strain-stiffen later than double networks prepared from randomly cross-linked networks with the same modulus, the onset of strain stiffening was surprisingly well-predicted by the molecular weight between cross-links targeted during the initial network synthesis.

Careful consideration of the molecular mechanisms underlying the modulus and strain stiffening behavior of polymer networks and double-network elastomers provides insight into the origins of this result. As established in the first half of the 20th century,^54−58^ the modulus of a polymer network, which is measured at low strain, reflects the number of elastically effective strands per unit volume (eq 1). While this modulus is often used to calculate an average molecular weight between cross-links (eq 2), doing so requires assuming that all of the strands in the network are elastically effective. In real networks, however, where defects such as loops, dangling ends, and densely cross-linked regions that do not deform with the rest of the network reduce the number of elastically effective strands per unit volume,^34,59^ this calculation overestimates the actual length of the network strands. The onset of strain stiffening in both single- and double-network elastomers, on the other hand, arises from the finite extensibility of the elastically effective strands^47,52,53,57,58^ and is thus much more directly related to their actual length. In this context, we interpret our results as follows: networks synthesized with the same target molecular weight between cross-links have similar average elastically effective strand lengths, regardless of network topology, and strain-stiffen at similar macroscopic extensions, yielding similar fitted values of *n*. The random networks have a lower modulus than the regular networks, however, because they have a higher defect content (arising from spatial heterogeneities that arise during polymerization and cross-linking) and thus a lower fraction of strands that are elastically effective. By contrast, networks with the same modulus have the same number of elastically effective strands per unit volume, but the strands that are elastically effective are longer in the regular networks (which have lower defect content), delaying the onset of strain stiffening and yielding larger fitted values of *n* for the regularly cross-linked samples.

We emphasize this point because polymer networks are often described simply in terms of their average molecular weight between cross-links, calculated from the modulus using eqs 1 and 2. As our experiments make clear, however, this value does not actually carry any information about the length of the elastically effective strands in the material. If the value of *M*_*x*_ calculated from the modulus gave a true “cross-link length”, then all networks with the same modulus—and thus the same value of *M*_*x*_—would be expected to strain-stiffen at the same point, which is clearly contradicted by our data. Thus, while *M*_*x*_ calculated by this method offers a useful proxy for a network’s modulus, it does not reflect the actual length of the elastically effective strands, and obtaining an accurate picture of the material’s molecular-scale features requires characterization of both the low- and high-strain behavior.^60^

We note that this interpretation of the random versus regular network behavior is most robust for ideal networks. Here, we prepared close-to-ideal regularly cross-linked networks by (1) using controlled polymerization methods to synthesize star polymer precursors with narrow dispersities and high end-group retention, (2) preparing the regularly cross-linked networks from tetrafunctional rather than difunctional polymers to maximize excluded volume effects, and (3) ensuring that the polymer concentrations during gelation were well above the overlap concentration (see Supporting Information). The gel fractions measured for the REG networks (see Supporting Information) do suggest that gel formation was largely complete, but the comparison of the experimental and theoretical moduli in Table 2 indicates that the REG networks still contained non-negligible defect content, and understanding the role of loop and dangling-end defects may be an interesting direction for future work. We also note that rheological characterization and Mooney–Rivlin plots suggest that the low-strain responses of some of the more loosely cross-linked networks may contain non-negligible contributions from entanglements and/or slow processes that may not fully relax at the experimental strain rates (see Supporting Information). The primary trends in Figure 5 hold even when these samples are omitted, indicating that the observed differences between the RAN and REG samples are not simply due to these effects. However, understanding the impacts of both entanglements and strain rates will be important for future work.

Beyond these points, there are a number of subtleties in the interpretation of the strain stiffening behavior that merit further investigation. It is not clear from the present data, for example, whether the difference in the strain stiffening behavior of regular and random networks with the same modulus arises from differences in the *average* length of the elastically effective strands, or whether it arises from differences in the lengths of the *shortest* elastically effective strands. In the affine deformation limit, the onset of strain stiffening should coincide with the shortest strands reaching their maximum extensibility. When junction points are allowed to relax, however, stress may be more evenly distributed across chains of different lengths.^61,62^ In this limit, the network may not strain-stiffen until the shortest percolating paths through the network reach their maximum extensibility,^63^ and because the percolating paths in a macroscopic sample contain a large number of chains, their strain stiffening behavior should reflect the average length of the elastically effective strands rather than the shortest. While MD simulations on double-network elastomers have indeed highlighted the role of percolating paths in determining the strain stiffening of double-network elastomers,^53^ the specific role of the strand length distribution has not been investigated, and experiments and simulations in which the strand length distribution is well-controlled and systematically varied will be critical for testing this prediction. Second, we note that at low strain the swelling-corrected stress of the double-network samples is systematically higher than that expected from the corresponding single networks (see Supporting Information). While some difference between the moduli of the single- and double-network samples is expected,^47^ entanglement with the second network may also make loop defects in the first network elastically effective, and the presence of the second network may restrict relaxation of the junction points, both of which would increase the moduli of the double-network samples. Understanding how double networks affect the full stress–strain responses of the materials beyond simply providing mechanisms for energy dissipation will be important for future work in this area. Finally, our work highlights the need for models of rubber elasticity that accurately account for differences in network topology and strand length distributions in the high strain limit. Here, we used the eight-chain model to analyze strain stiffening of the networks because it provides a simple molecular interpretation of the origin of this behavior.^52^ However, this model does not accurately reflect the topology of the networks and implicitly assumes that they are monodisperse and undergo affine deformation. As a result, it is not possible to directly map the fitted strand length, *n*, onto the actual degree of polymerization of the cross-link strands. While some progress has been made in developing theories and simulation models that better represent the structures of real polymer networks in the high-strain limit,^51,64−67^ further work is needed in this area.

While we focus here on understanding the fundamental features dictating strain stiffening in elastomeric networks, our results also suggest new avenues for independently controlling the modulus and strain stiffening of polymer materials by changing the network topology. The strain stiffening behavior of polymer networks should be controllable by changing the length of the elastically effective strands. The modulus of the networks, on the other hand, should be independently controllable by changing the defect content of the networks and, thus, the fraction of elastically effective strands. These directions are complementary to those that focus on decoupling modulus and strain stiffening by changing the architecture of the cross-links themselves, such as in bottlebrush elastomers.^68^ Further experiments on regularly cross-linked networks, which allow the strand length and defect content to be controlled in ways that are impossible in randomly cross-linked materials, will be critical for further elucidating the impact of network topology on strain stiffening and informing design rules for materials with precisely targeted low- and high-strain behavior.

## Conclusion

We synthesized a series of randomly and regularly cross-linked butyl acrylate networks and used a double-network strategy to investigate their high-strain behavior. We found that changing the network type significantly impacted the onset of strain stiffening in these materials, with double-network elastomers prepared from regular networks exhibiting significantly later strain stiffening than double-network elastomers prepared from random networks with the same modulus. We attribute this behavior to the difference in the topologies of the networks. Random networks have many short strands, which lead to earlier strain stiffening, but a lower fraction of elastically effective strands, which reduces the modulus. By contrast, regular networks with the same modulus have primarily longer strands, which lead to delayed strain stiffening, but most of these strands are elastically effective, maintaining a higher modulus. These results highlight the different origins of the low- and high-strain behavior of polymer networks and the need for robust theoretical models of their high-strain behavior that account for variations in strand length and connectivity. Our results also suggest new avenues for independently tuning the low- and high-strain behavior of flexible polymer networks, which we anticipate will enable new frontiers in materials for wearable sensors,^69^ shape memory polymers,^70^ and other applications requiring networks to function in the high-strain limit.^71^ Together, this work provides both fundamental and applied insight into the strain stiffening behavior of polymer networks and suggests that regular networks will be an important platform for further understanding the origins of networks’ responses across a wide range of strains.

## Supporting Information Available

Detailed synthetic conditions for polymer and network synthesis, supplemental characterization data (NMR, MALDI, SEC, overlap concentrations, rheology, and repeatability of tensile measurements), and details of the model used to analyze the tensile data (PDF)
