# Supplementary material for: Effect of Cross-Link Homogeneity on the High-Strain Behavior of Elastic Polymer Networks
Source: Macromolecules. 2024 May 8;57(10):4670–9. doi: 10.1021/acs.macromol.3c02565 (PMC11140753; doi:10.1021/acs.macromol.3c02565)
Supplement: Supplementary file 1 — ma3c02565_si_001.pdf [file ma3c02565_si_001.pdf]

Supporting Information: Effect of Crosslink Homogeneity on the High Strain Behavior of Elastic Polymer Networks

Victoria A. Kong, Thomas A. Staunton, and Jennifer E. Laaser*

Department of Chemistry, University of Pittsburgh, Pittsburgh, PA

E-mail: j.laaser@pitt.edu

Phone: (412)383-0125

Supplemental Methods

Materials

Butyl acrylate (BA, 99+%, stabilized, ACROS Organics) and 1,6-hexanediol diacrylate (HDD, 80%, technical grade, Sigma-Aldrich) were passed through activated neutral alumina to remove inhibitor and sparged with argon before use. Benzoyl peroxide (BPO, 98%, reagent grade, Sigma-Aldrich), copper (II) bromide ($CuBr_2$, 99.999%, Sigma-Aldrich), copper (0) wire, ethanol (EtOH, 100% , Fisher Scientific), N,N-dimethylformamide (DMF, 99.5%, Sigma-Aldrich), anhydrous dimethyl sulfoxide (DMSO, 99.9%, Fisher Scientific), N-methyl-1,3-diaminopropane (MPDA, Fisher Scientific), tris[2-(dimethylamino)ethyl]amine (ME_6TrEN, 97%, Sigma-Aldrich), 3,6-dioxa-1,8-octanedithiol (DODT, 97.0+%, Fisher Scientific), and pentaerythritol tetrakis(2-bromoisobutyrate) (Tetra-BriB, Sigma-Aldrich) were used as received. All reactions were carried out under air free conditions unless otherwise

noted.

Polymer Synthesis

Synthesis of Randomly-Crosslinked Networks

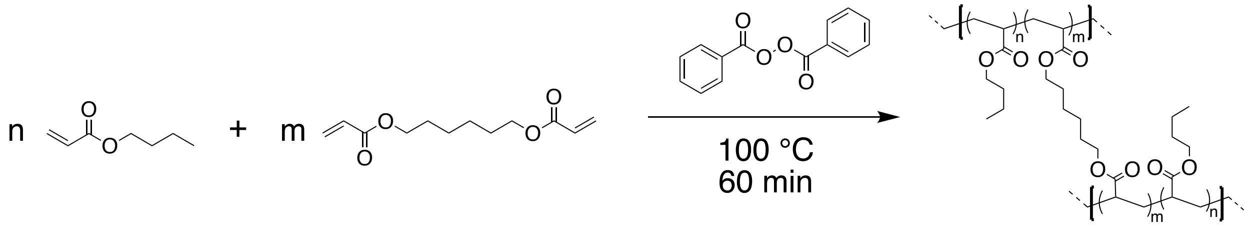

Scheme S1: Synthesis of randomly crosslinked butyl acrylate networks

Randomly crosslinked networks were synthesized by free-radical polymerization of n-butyl acrylate (monomer) and hexanedioldiacrylate (crosslinker) in toluene. In a typical synthesis targeting a network with a molecular weight between crosslinks (M_x) of 5 kg/mol, butyl acrylate (10 mL, 8.9 g, 69 mmol, 80 eq) and hexanedioldiacrylate (199 μL, 197 mg, 0.87 mmol, 1 eq) were combined in an argon-purged vial. In parallel, 10 mL of a 0.07 M solution of benzoyl peroxide (0.8 eq) in toluene was sparged with argon for 10 minutes. This solution was then injected into the BA/HDD mixture, after which the mixture was vortexed and injected into a circular PTFE-lined mold (Fig. S1). The mold was sealed and placed into an oven at 100 °C for one hour to polymerize the reaction mixture. After polymerization, the samples were removed from the oven, released from the mold, and placed gently into a 3:1 methanol:acetone bath to allow unreacted monomer to diffuse out from the network. The bath was changed daily for 5 days, after which the network was allowed to dry on the benchtop or in a warm (45 °C) oven under ambient pressure for two days. Once visible signs of residual solvent (e.g. opaque white spots in the sample) were no longer observed, the network was placed in a 50 °C oven under vacuum to make sure the network was completely dry, yielding the final network as a clear, colorless, rubbery material.

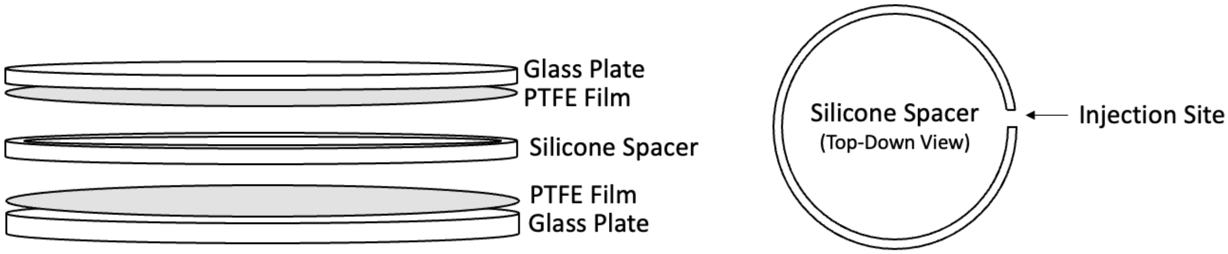

Figure S1: Schematic of the injection mold used in the synthesis of randomly crosslinked networks. Reaction mixtures were injected into the gap in the 1.3 mm thick silicone spacer, after which the molds were sealed using teflon tape, high temperature electrical tape, and binder clips around the perimeter of the mold.

Samples targeting other crosslinking densities were synthesized following the same general procedure, with the amount of crosslinker needed calculated using

$$n_{HDD} = \frac{M_0 n_{BA}}{2M_x} \tag{1}$$

where M_0 is the molecular weight of the monomer, n_{BA} is the moles of monomer, M_x is the targeted molecular weight between crosslinks, and n_{HDD} is the requisite moles of hexanediol diacrylate crosslinker. The reagent quantities used for all samples reported in this work are summarized Table S1.

Table S1: Reagents used in the synthesis of randomly crosslinked networks

Sample	Monomer[a] [g]	[mL]	Crosslinker[b] [mg]	[μL]	Initiator[c] [mg]	Solvent[d] [mL]
RAN_5	8.9	10	197	199	168	10
$RAN_{8.8}$	8.9	10	113	114	168	10
RAN_{10}	8.9	10	98.7	99.7	168	10
RAN_{15}	8.9	10	65.8	66.5	168	10
RAN_{20}	8.9	10	49.4	49.9	168	10
RAN_{25}	8.9	10	39.5	39.9	168	10
RAN_{30}	8.9	10	32.9	33.2	168	10

[a)]n-butyl acrylate, inhibitor-free; [b)]hexanediol diacrylate, inhibitor free; [c)]benzoyl peroxide; [d)]toluene

Synthesis of Tetrafunctional Poly(n-butyl acrylate) Star Polymers

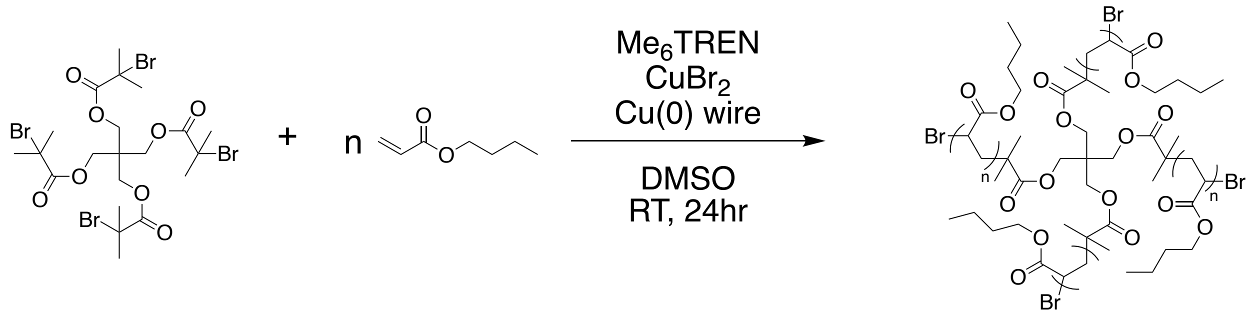

Scheme S2: Synthesis of tetrafunctional poly(n-butyl acrylate) star polymers

Tetrafunctional poly(n-butyl acrylate) star polymers used in the synthesis of regular networks were prepared by SET-LRP, as shown in Scheme S2. To activate the Cu(0) source for the catalyst, copper wire was first wrapped around a stir bar and placed in a side-arm round bottom flask under Ar(g). The flask was filled with enough degassed H_2SO_4 to cover the stir bar. The solution was stirred vigorously under flow of Ar for 10-15 minutes, after which the copper turned a shiny, light orange color. The H_2SO_4 was then removed from the flask and the flask and stir bar were rinsed with degassed MeOH at least three times to remove any remaining acid. The flask and stir bar were finally dried under flow of Ar(g) and stored under Ar prior to use. In a typical synthesis targeting a 15 kg/mol polymer, the deactivator, $CuBr_2$ (15.7 mg, 0.1 eq.), was added to the flask containing the Cu-wrapped stir bar under flow of argon. A degassed mixture of n-butyl acrylate monomer (13.7 g, 156 eq.), Me_6TREN ligand (37.3 mg, 0.24 eq.), and anhydrous dimethyl sulfoxide (1:1 v:v relative to monomer) was cannulated into the flask, after which the tetra-BriB initiator (0.5 g, 1 eq.) was added under flow of Ar. The reaction mixture was stirred at high speed (600 rpm) at room temperature for 24 hours, during which time it changed from a transparent, low-viscosity blue/green solution to a green, cloudy, higher viscosity liquid as the polymers began to phase separate from the DMSO solvent. After 24 hours, the reaction was opened to air to terminate polymerization. The polymer was then precipitated into a cold 9:1 ethanol:water

mixture and passed through a silica column to remove residual $CuBr_2$. The purified polymer was dried under vacuum at 50 °C until all residual solvent was removed (as analyzed by ^{1}H NMR), yielding the polymer as a clear, colorless, viscous material. (Yield: 54%. ^{1}H NMR (500MHz, $CDCl_3$)) δ: 0.93 (bt, 3H), 1.37 (bs, 2H), 1.60 (bs, 2H), 1.90 (bs, 1H), 2.3 (bs, 1H), 4.03 (bm, 2H) ppm. MALDI-TOF: M_w: 15 kg/mol, M_n: 14 kg/mol, Đ: 1.03)

Following this general procedure, the other tetrafunctional star polymers were synthesized using the reagent ratios shown in Table S2. Polymers are labeled BA_x, where the subscript indicates the total molecular weight of the star polymer in kg/mol. For all polymers, MALDI and SEC data (Figs. S3-S4) were monomodal, indicating minimal bimolecular coupling; for polymers with low enough molecular weight to allow MALDI analysis (BA_{14} and BA_{20}), MALDI spectra also suggested minimal end-group loss *via* disproportionation (Fig. S3).

Table S2: Reagents used in the synthesis of BA_{14}, BA_{40}, BA_{52}, and BA_{77} tetrafunctional star polymers

Sample	^{a}Monomer			^{b}Initiator		^{c}Ligand			^{d}Deactivator		^{e}Solvent	^{f} Target M_n
	[g]	[mL]	[mol equiv.]	[g]	[mol equiv.]	[mg]	[μL]	[mol equiv.]	[mg]	[mol equiv.]	(mL)	(kg/mol)
BA_{14}	13.7	15.4	156	0.5	1	37.3	43.2	0.24	15.7	0.10	15.4	20
BA_{40}	68.3	76.7	390	1	1	74.5	86.5	0.24	31.4	0.10	76.7	50
BA_{52}	41.0	46.0	468	0.5	1	37.3	43.2	0.24	15.7	0.10	46.0	60
BA_{77}	62.3	70.0	712	0.5	1	35.7	39.7	0.23	14.8	0.097	70.0	91

a) n butyl acrylate, inhibitor-free; b) Pentaerythritol tetrakis(2-bromoisobutyrate); c) Tris[2-(dimethylamino)ethyl]amine; d) $CuBr_2$; e) Dimethyl sulfoxide (anhydrous); f) Calculated assuming 100% conversion (note: phase separation during synthesis prevented measurement of actual final conversion)

Table S3: Summary of tetrafunctional poly(n-butyl acrylate) star polymers

$Polymer_{M_x}$	M_n [kg/mol]	M_w [kg/mol]	Đ
$^a BA_{14}$	14	15	1.03
$^a BA_{40}$	40	41	1.03
$^b BA_{52}$	52	59	1.13
$^b BA_{77}$	77	89	1.16

[a] Analyzed by size exclusion chromatography (TOSOH ECO-SEC; eluent: THF; temperature: 40 °C; absolute molecular weight analysis using Wyatt Dawn8+ light scattering detector with $dn/dc = 0.067$, [b] Analyzed by MALDI-TOF (matrix: IAA/Na^+TFA); method:LP5-50kDa)

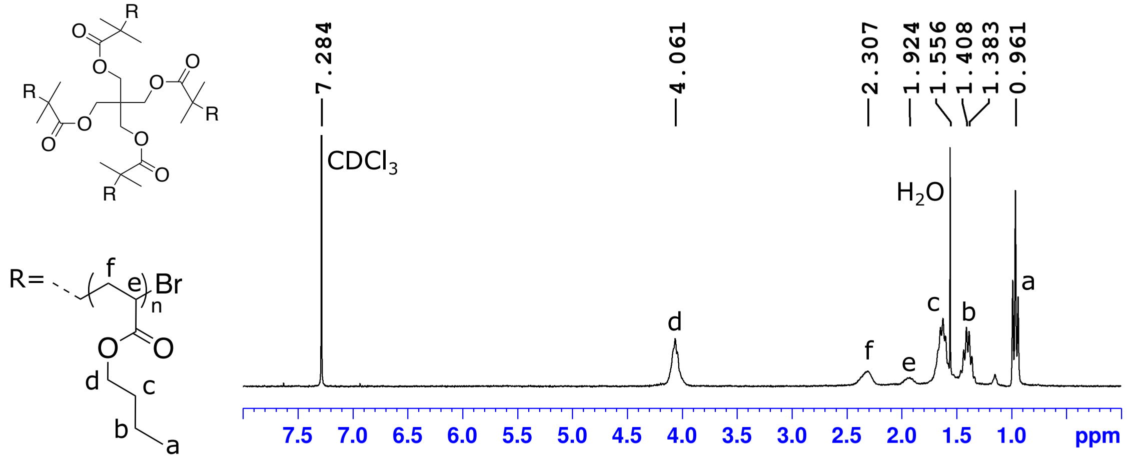

Figure S2: H-NMR spectrum (500MHz, $CDCl_3$) of BA_{40} tetrafunctional poly(n-butyl acrylate) star polymers).

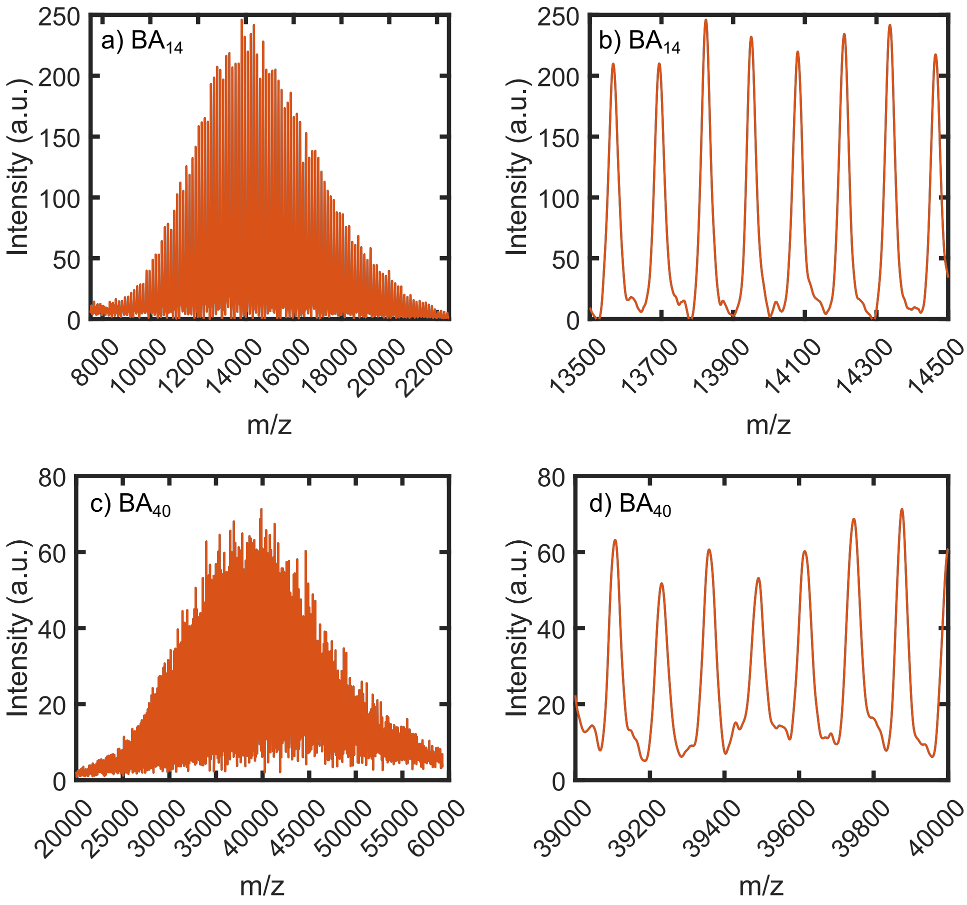

Figure S3: MALDI spectra of (a,b) BA_{14} and (c,d) BA_{40} tetrafunctional poly(n-butyl acrylate) star polymers.

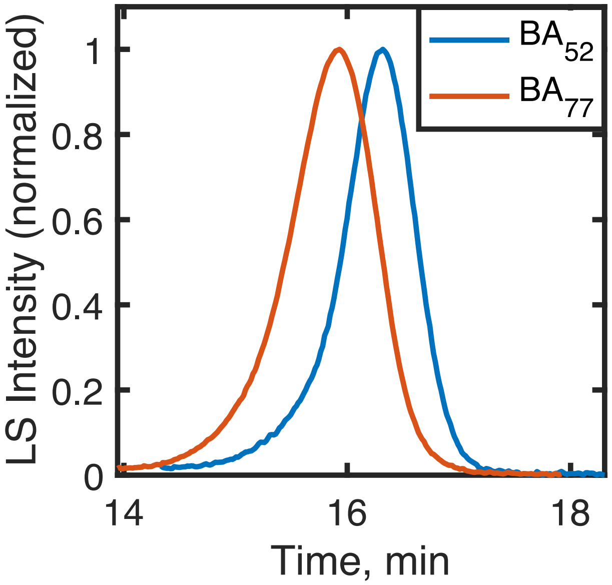

Figure S4: SEC traces of BA_{52} and BA_{77} tetrafunctional poly(n-butyl acrylate) star polymers.

Synthesis of Regularly-Crosslinked Networks

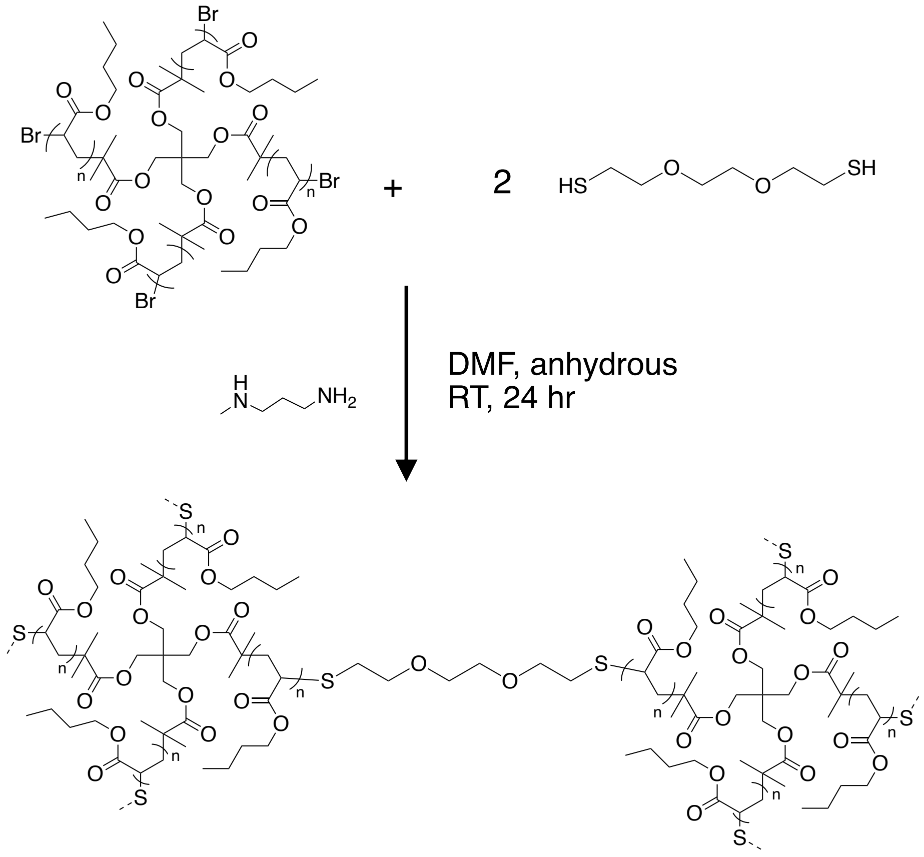

Scheme S3: Synthesis of regular networks via thiol-bromine click reactions of tetrafunctional star polymers and difunctional crosslinkers

Regularly-crosslinked networks were synthesized by crosslinking the tetrafunctional star polymers using a thiol-bromine click reaction. In a representative synthesis of the network with M_x = 26 kg/mol, the precursor tetrafunctional star polymer (BA_{52}, 4.25g, 0.08 mmol polymer, 1 eq.), DODT (0.0280g, 0.15 mmol, 2 eq.), MPDA (36.8 mg, 43.6 μL, 5.2 eq.) and anhydrous DMF (16.94 mL) were brought into a glovebox. The polymer and crosslinker were combined, dissolved in 70 % of the total DMF volume (approx. 12 mL), and degassed in the glovebox antechamber to remove bubbles formed during mixing. The MPDA was dissolved in the remaining 30% of the DMF, after which both solutions were combined in a falcon tube and gently inverted to mix. The mixture was then poured into a teflon mold, covered with a petri dish to protect from dust, and left in the glovebox to react for 24 hours. After 24 hours,

the samples were removed from the glovebox, gently released from the molds, weighed, and measured. The DMF was then removed by soaking the samples in a 3:1 methanol:acetone bath. The solvent bath was changed daily for 5 days, after which the sample was removed from the solvent and allowed to dry. To speed up drying, the network was placed in a warm (45 °C) oven under ambient pressure for two days. Once visible signs of residual solvent (e.g. opaque white spots) were no longer observed, the sample was transerred to a 50 °C oven and dried under vacuum overnight to ensure that all solvent was removed, yielding the network as a clear, colorless, rubbery sample.

Preparation of Double Networks

To synthesize double networks, the network of interest was submerged in a cold (2-4 °C) solution of n-butyl acrylate monomer (1 eq.), hexanedioldiacrylate crosslinker (0.1 meq.), and benzoyl peroxide initiator (0.1 meq.) in an argon-flushed chamber. The networks were held at 2-4 °C and allowed to swell to equilibrium (minimum 2 hours), after which they were removed from the monomer/crosslinker solution under flow of Ar and gently blotted to remove excess monomer. Samples were then placed between two PTFE sheets with a silicone spacer and sandwiched between glass plates. The mold was sealed and placed in an oven at 100 °C for an hour to initiate polymerization of the second network. After one hour, the samples were removed from between the glass plates and placed back in the 100 °C oven overnight to evaporate unreacted monomer and crosslinker. Networks were dried under ambient pressure to avoid formation of bubbles, yielding the final double network as a clear and colorless rubbery material.

Uniaxial Extension Tests

Tensile samples were cut from the fully dried polymer networks using a custom micro dog-bone die cutter, with dimensions shown in Fig. S5. The design of the die cutter is adapted from ASTM standard D638 type IV.[S1] The resulting tensile samples were then loaded onto

either (1) an ADMET eXpert 5601-2C or (2) Material Testing Systems Insight Dual Transformer 820 XFMR-DUAL mechanical tester. The modulus of each sample was calculated using a linear fit to the stress-strain data at extension ratios below 1.2 and averaged over 3-5 samples.

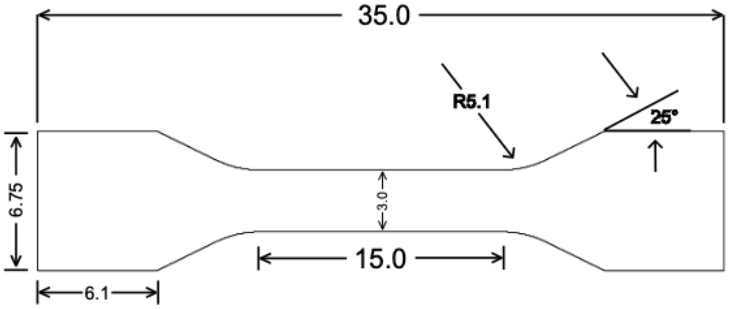

Figure S5: Dimensions of micro dog-bone specimens used for uniaxial tensile tests. Dimensions are in mm.

Supplemental Results

Overlap Concentrations of Star Polymers

The overlap concentrations, c^*, of the star polymers used in the regular network syntheses were determined from their intrinsic viscosities, $[\eta]$, using

$$c^* \approx \frac{1}{[\eta]} \tag{2}$$

Intrinsic viscosity measurements were conducted in the same solvent used for network synthesis (anhydrous DMF) using Ubbelohde viscometers thermostatted at 25 °C. The intrinsic viscosities of the polymers, and the corresponding overlap concentrations, are summarized in Table S4. As seen in this table, the overlap concentrations of the polymers were at least a factor of two lower than the concentration of polymer used during the REG network syntheses (approx. 20 wt%), indicating that all polymers were well above their overlap concentrations

during gelation.

Table S4: Intrinsic Viscosities and Overlap Concentrations of Star Polymers in Anhydrous Dimethylformamide at 25 °C

Sample	Intrinsic Viscosity, $[\eta]$ [dL/g]	Overlap Concentration, c^* [mg/mL]
BA_{14}	0.102	98.2
BA_{40}	0.178	56.1
BA_{52}	0.183	54.7
BA_{77}	0.241	41.6

Gel and Sol Fractions of Single Networks

Small duplicate single network samples were weighed before and after the washing step described in the previous section to determine the fraction of the precursors (monomer/crosslinker or star polymer) covalently linked into the network. The resulting sol and gel fractions are tabulated in Table S5. As seen in this table, the gel fraction for the RAN networks was somewhat lower than that of the REG networks, consistent with the lower moduli (corresponding to a lower network quality) measured for the RAN samples. We note that REG_{20} was omitted from the sol/gel analysis due to limitations in the amount of polymer available to synthesize the duplicate sample, but is expected to have sol & gel fractions similar to those of the other REG samples.

Table S5: Sol and Gel fractions of Single Networks

	Mass Fraction	
Sample	sol	gel
RAN_5	0.17	0.83
$RAN_{8.8}$	0.21	0.79
RAN_{10}	0.21	0.79
RAN_{15}	0.27	0.73
RAN_{20}	0.34	0.66
RAN_{25}	0.32	0.68
REG_7	0.06	0.94
REG_{26}	0.07	0.93
REG_{38}	0.07	0.93

Small-Amplitude Oscillatory Shear Rheology

Small amplitude oscillatory shear rheology was used to quantify the relaxation timescales of the randomly-crosslinked single network samples. Duplicate samples of all randomly-crosslinked materials were prepared using the same conditions as used for the tensile samples. Small-amplitude oscillatory shear measurements were then carried out using an Anton Paar MCR-302 rheometer with a 25 mm sandblasted parallel plate geometry at a strain amplitude of 0.1%. The dynamic moduli of the materials are shown in Fig. S6. As seen in this figure, the RAN_5, $RAN_{8.8}$, and RAN_{10} samples all exhibited a characteristic elastic plateau in G' at frequencies below approximately 1 rad/s. The RAN_{15}, RAN_{20} and RAN_{25} samples, on the other hand, did not exhibit a characteristic elastic plateau, suggesting that entanglements dominate the mechanical response of the samples at the experimentally-relevant strain rates (see "Contributions of Entanglements", below). Amplitude sweeps at 1 rad/s (Fig. S7) confirm that the strain amplitude used in the frequency sweeps was within the linear regime. Usable rheology data could not be obtained for the regularly-crosslinked materials due to the limited amount of polymer remaining after preparation of the tensile samples, but Mooney-Rivlin plots suggest that these samples were not entangled and should exhibit relaxation behavior similar to that of RAN_5-RAN_{10}, as discussed in more detail, below.

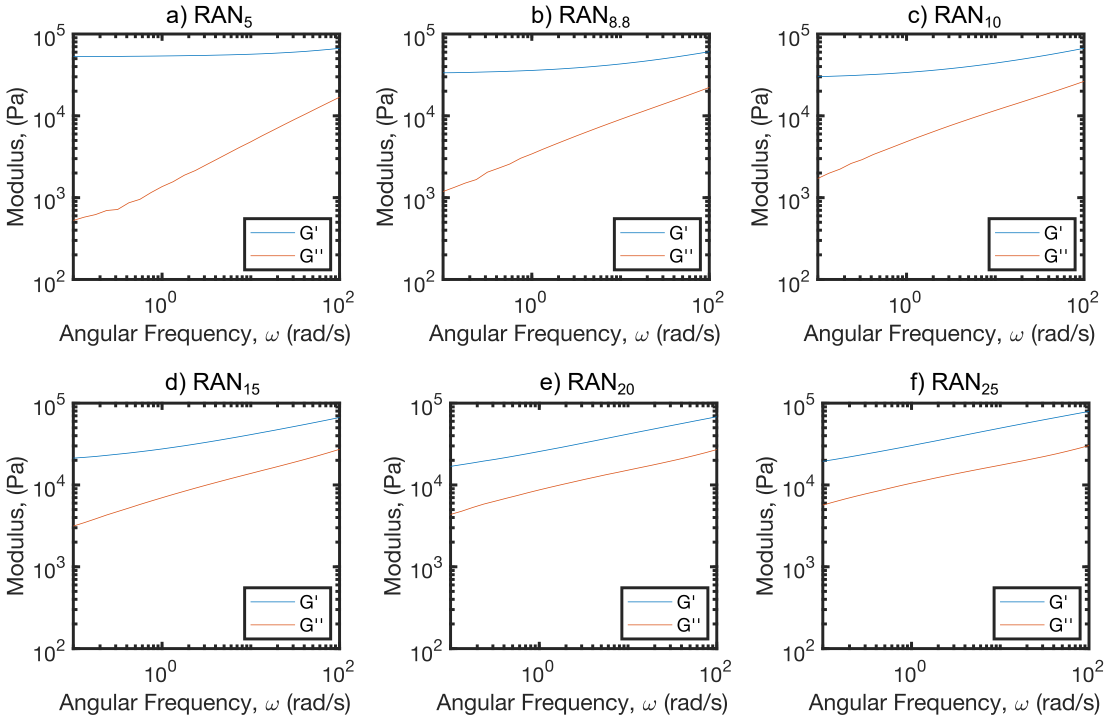

Figure S6: Frequency sweeps of duplicate RAN samples carried out at a strain amplitude of 0.1%.

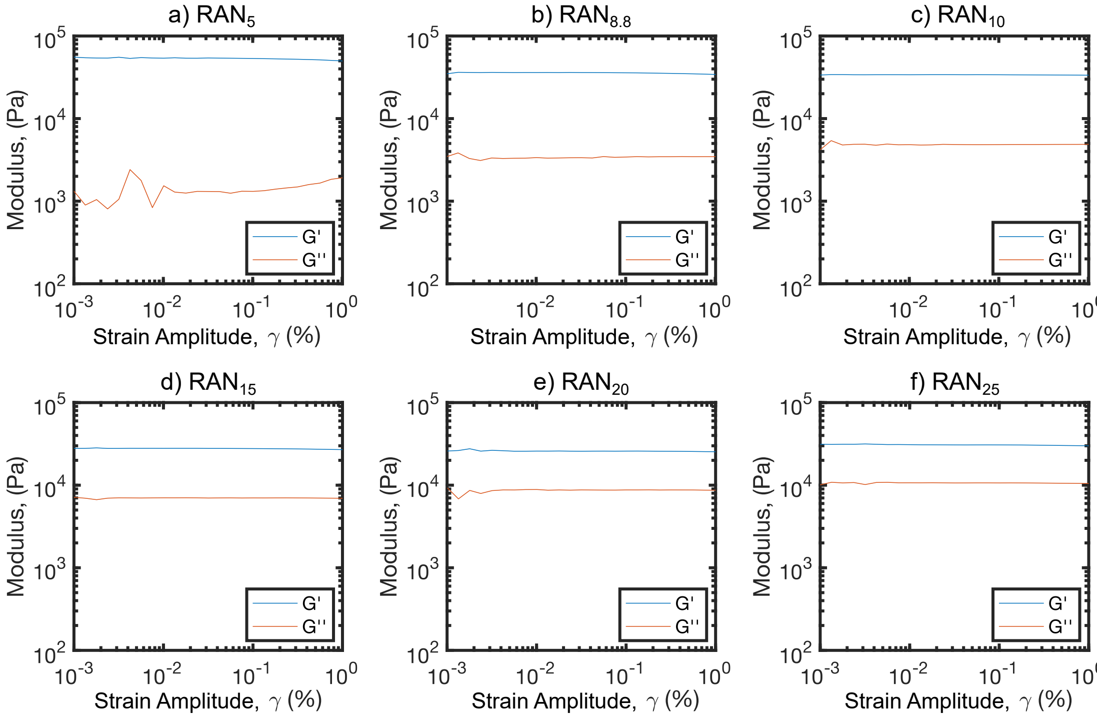

Figure S7: Amplitude sweeps of duplicate RAN samples carried out at a frequency of 1 rad/s.

Contributions of Entanglements

Although the targeted M_x values for the majority of the samples reported in this work were greater than the entanglement molecular weight of poly(n-butyl acrylate) ($M_e \approx 26 - 32$ kg/mol, ref. S2), rheology measurements suggested that the responses of the most lightly-crosslinked samples may be dominated by entanglements at the strain rate used in the tensile measurements. To quantify the contributions of entanglements to the measured responses of both the random and regular single networks, the tensile data was fit to the Rubinstein-Panyukov model.[S3] In this model, the mechanical response of an entangled network is predicted to follow

$$\sigma^* = \frac{\sigma}{\lambda - \lambda^{-2}} = G_c + \frac{G_e}{\lambda - \lambda^{1/2} + 1} \tag{3}$$

where G_c is the portion of the modulus attributable to covalent crosslinks and G_e is the portion of the modulus attributable to entanglements. Mooney-Rivlin plots depicting the reduced stress (σ^*) as a function of $1/\lambda$ and the associated fits are shown in Figs. S8 and S9, and the fit parameters G_c and G_e are tabulated in Table S6. As seen in this data, the tensile responses of the RAN_5, $RAN_{8.8}$, RAN_{10}, and all REG networks appear to be dominated by the covalently crosslinked network (as evidenced by flat Mooney-Rivlin plots and values of $G_c > G_e$) at low strain, while the tensile responses of the RAN_{15}, RAN_{20}, and RAN_{25} networks appear to be have significant contributions from entanglements, consistent with the rheology data shown in the preceding section.

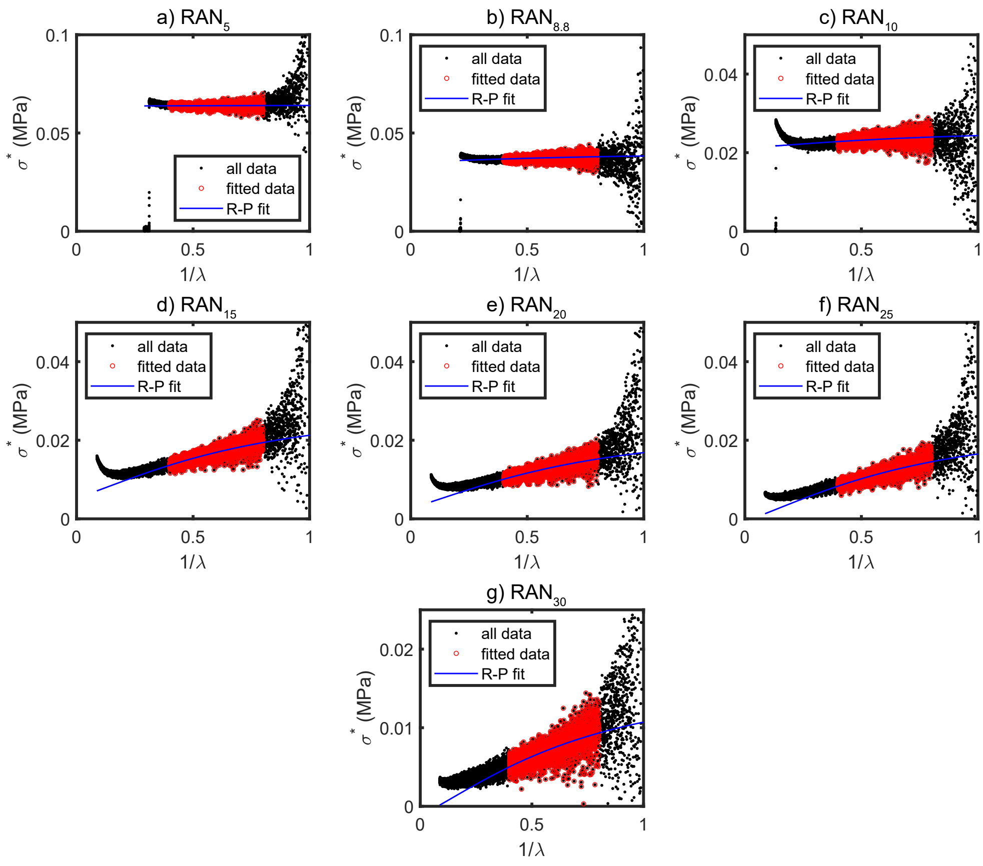

Figure S8: Mooney-Rivlin plots of RAN samples, with fits to Equation 3 for data between $0.4 < 1/\lambda < 0.8$.

Table S6: G_c and G_e Values Obtained from Fits to Eqn. 3

Sample	G_c (MPa)	G_e (MPa)
RAN$_5$	0.064	0.000
RAN$_{8.8}$	0.035	0.003
RAN$_{10}$	0.021	0.003
RAN$_{15}$	0.005	0.016
RAN$_{20}$	0.003	0.014
RAN$_{25}$	-0.001*	0.017
RAN$_{30}$	-0.001*	0.012
REG$_7$	0.073	-0.003*
REG$_{20}$	0.040	0.004
REG$_{26}$	0.022	0.008
REG$_{38}$	0.020	0.004

* Negative fit values are non-physical and were indistinguishable from 0.

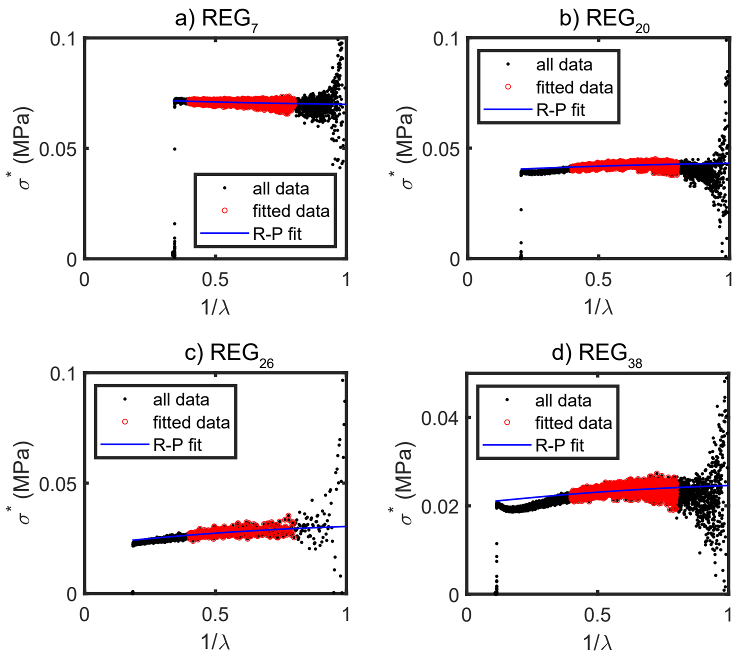

Figure S9: Mooney-Rivlin plots of REG samples, with fits to Equation 3 for data between $0.4 < 1/\lambda < 0.8$.

Comparison of SN and DN Data

To facilitate direct comparison of the responses of the single and double networks for each sample, tensile data were swelling-corrected using

$$\lambda_{eff} = \lambda \cdot \lambda^* \tag{4}$$

and

$$\sigma_{eff} = \sigma_{eff} = \sigma_N / \phi_{SN}^{2/3} \tag{5}$$

as has been reported previously.[S4] The swelling-corrected data is presented in Figs. S10-S12. As seen in this data, the stress in the double network samples was approximately twice that expected from the single network samples, suggesting that formation of the second network may either increase the number density of elastically effective strands in the first network (e.g. by transforming previously elastically-ineffective loops into elastically-effective strands) or by restricting junction point relaxation such that deformation is closer to affine.

For samples in which non-negligible strain stiffening was observed in both the single and double network samples, however, the inflection point in the data indicating the onset of strain stiffening appears at approximately the same strain (Fig. S12), indicating that the double network samples do accurately reflect the strain-stiffening properties of the networks from which they were formed.

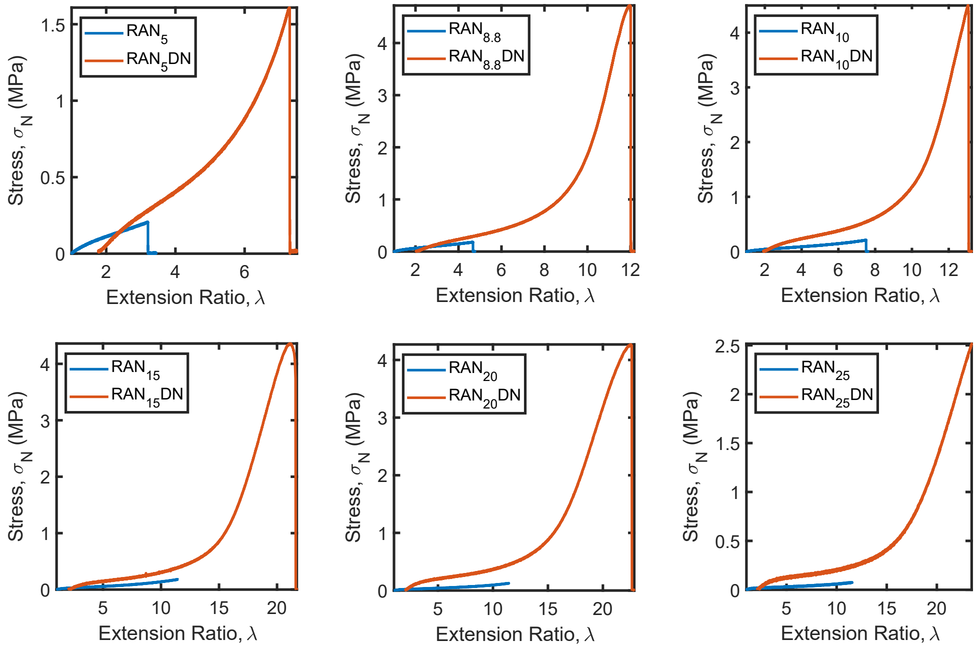

Figure S10: Comparison of single- and double- network tensile data for randomly-crosslinked networks. Double-network tensile data is corrected according to Eqns. 4 and 5 to reflect the stress and strain on the underlying single network.

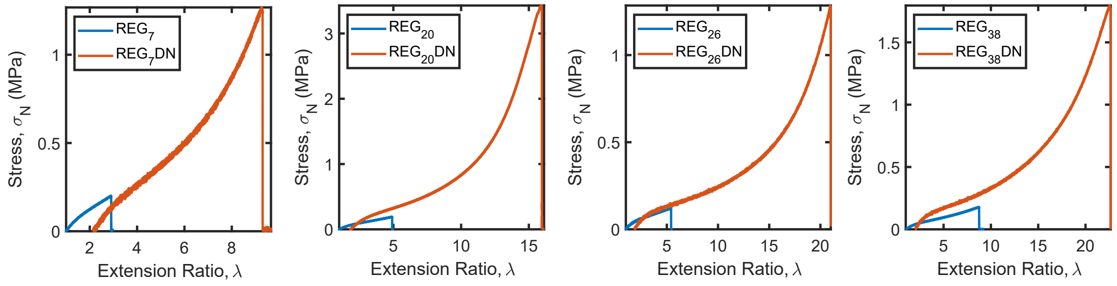

Figure S11: Comparison of single- and double- network tensile data for regularly-crosslinked networks. Double-network tensile data is corrected according to Eqns. 4 and 5 to reflect the stress and strain on the underlying single network.

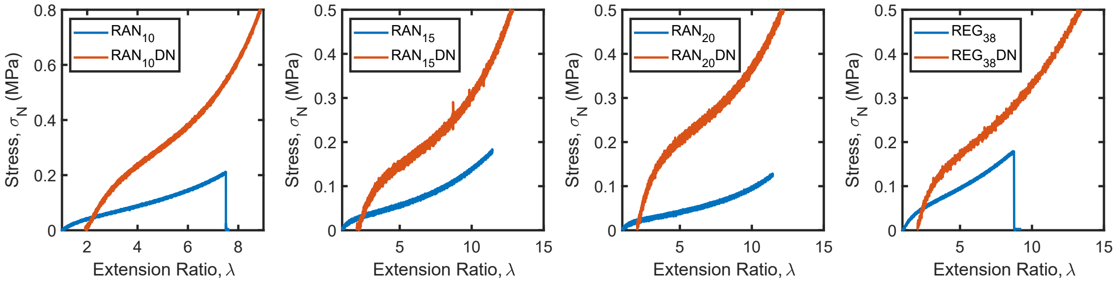

Figure S12: Comparison of single- and double- network tensile data for (a) RAN$_{10}$, (b) RAN$_{15}$, (c) RAN$_{20}$, and (d) REG$_{38}$, scaled to highlight the onset of strain stiffening. Double-network tensile data is corrected according to Eqns. 4 and 5 to reflect the stress and strain on the underlying single network.

Data Smoothing

As noted in the captions of Figures 1, 2, and 4 in the main text, and Figures S14-S15 in this Supporting Information, data for single network samples was smoothed using a Fourier filter prior to plotting to remove random noise due to electrical line interference (nominal frequency 60 Hz) and background vibrations in the laboratory. This procedure was helpful to minimize overlap between the plots of the tensile curves of the single network samples at low strain, but was not necessary for the double network samples, where the force was higher and the background noise constituted a much smaller percentage of the measured response. A representative trace showing the raw and smoothed data for RAN$_{25}$ is shown in Figure S13; as shown in this figure, this procedure did not change the shapes of the reported tensile curves. We note that smoothing was applied only for plotting purposes; all data was analyzed using the un-smoothed original data.

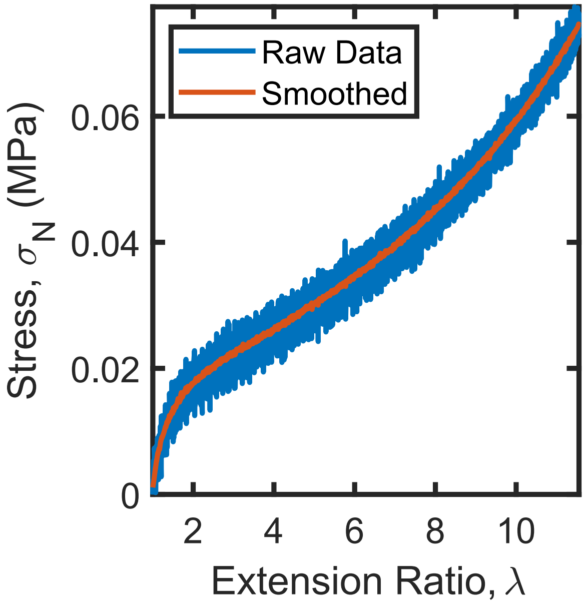

Figure S13: Raw data (blue) and smoothed data (red) for a representative stress-strain curve for RAN_{25}.

Measurement Repeatability

Tensile tests were carried out on 2-5 dogbones cut from the same bulk sample of each network. While the strain-at-break varied somewhat from replicate to replicate, the shapes of the stress-strain curves were generally repeatable, with all replicates of the same sample having similar moduli and onsets of strain stiffening (Figs. S14-S17).

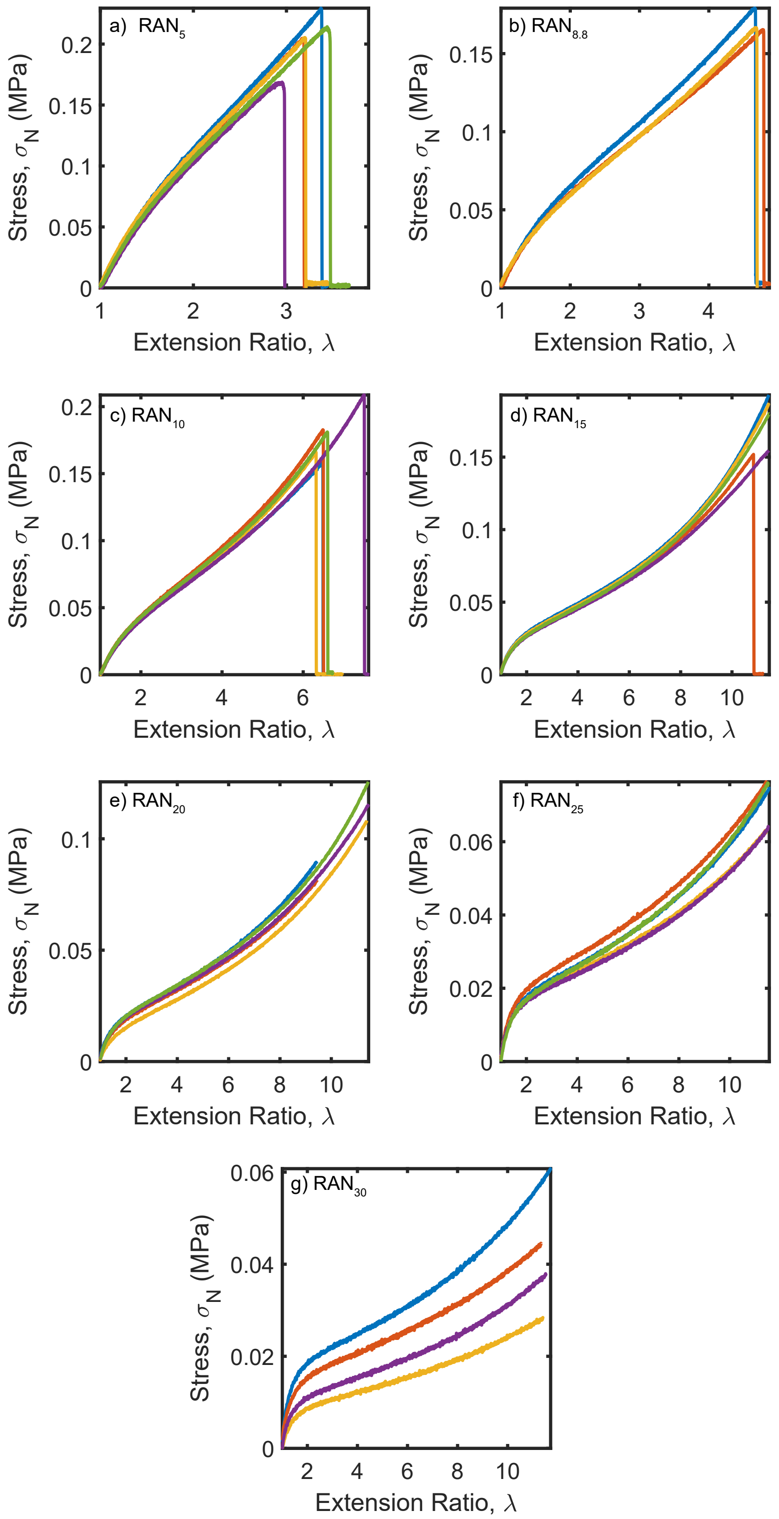

Figure S14: Stress-strain curves measured in repeated measurements of (a) RAN_5, (b) $RAN_{8.8}$, (c) RAN_{10}, (d) RAN_{15}, (e) RAN_{20}, (f) RAN_{25}, (g) RAN_{30}. Plotted data were smoothed using a Fourier filter to remove instrument noise and minimize overlap between the traces, as described above.

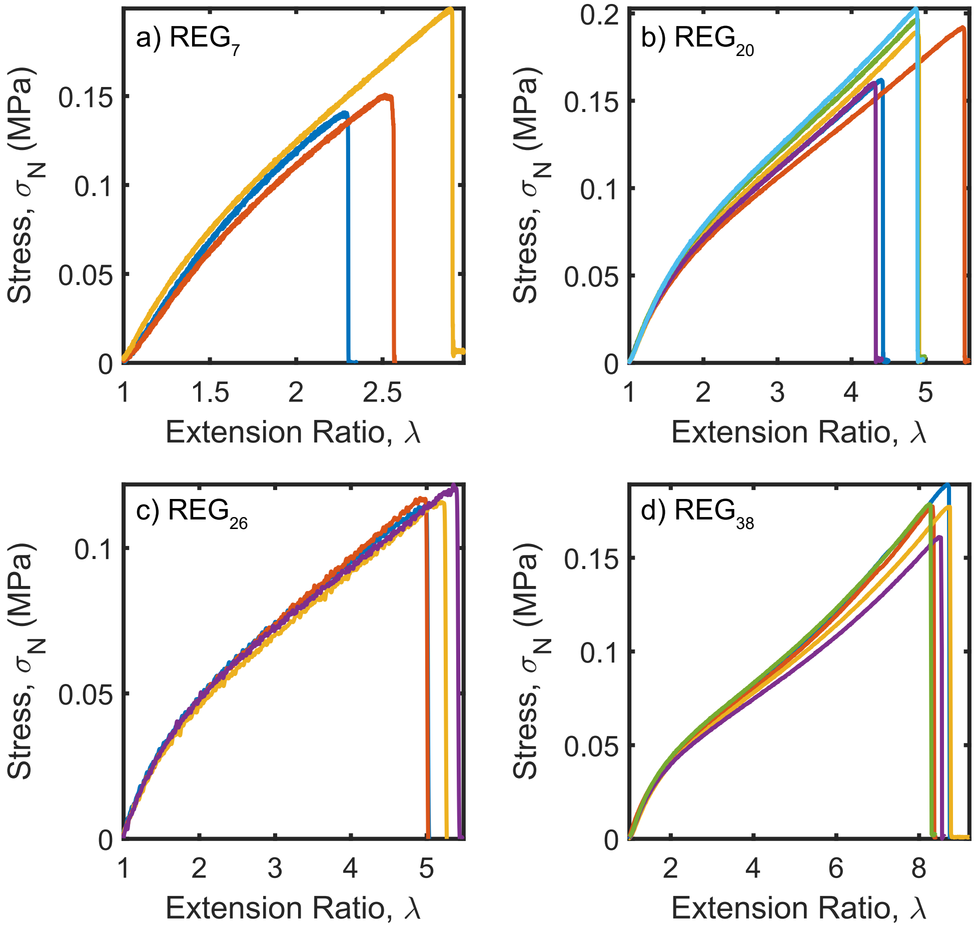

Figure S15: Stress-strain curves measured in repeated measurements of (a) REG_7, (b) REG_{20}, (c) REG_{26}, (d) REG_{38}. Plotted data were smoothed using a Fourier filter to remove instrument noise and minimize overlap between the traces, as described above.

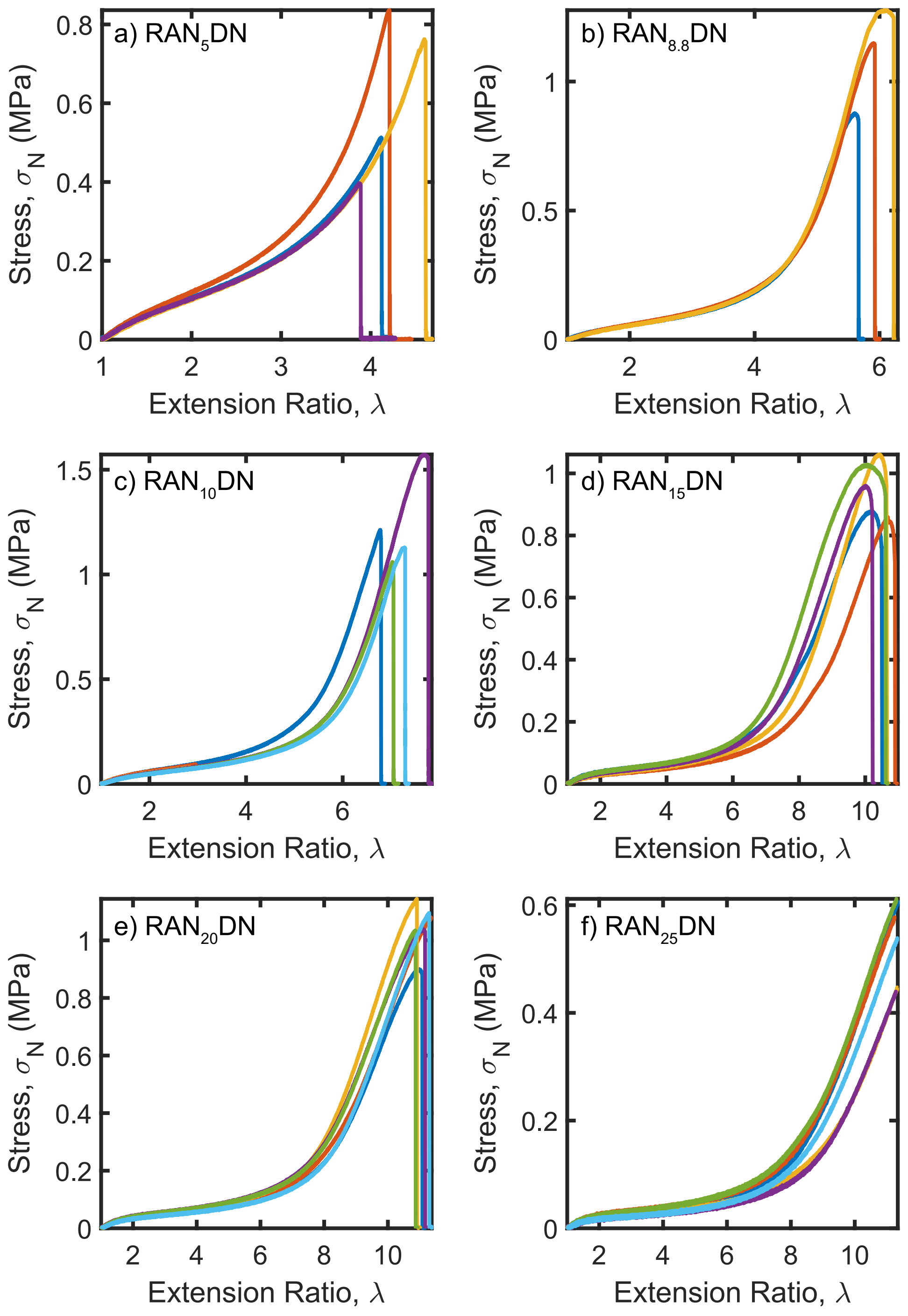

Figure S16: Stress-strain curves measured in repeated measurements of (a) RAN_5DN, (b) $RAN_{8.8}DN$, (c) $RAN_{10}DN$, (d) $RAN_{15}DN$, (e) $RAN_{20}DN$, (f) $RAN_{25}DN$.

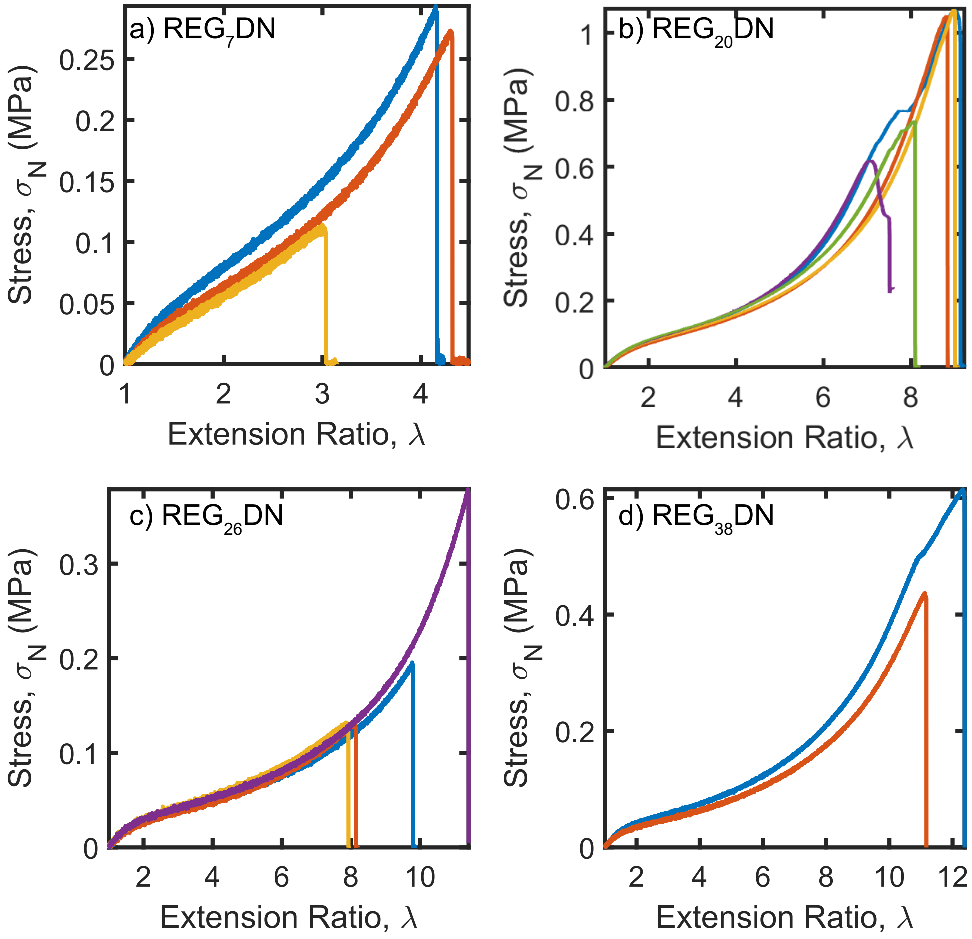

Figure S17: Stress-strain curves measured in repeated measurements of (a) REG_7DN, (b) $REG_{20}DN$, (c) $REG_{26}DN$, (d) $REG_{38}DN$.

Supplemental Analysis

Eight-Chain Model with Pre-Swelling

As noted in the main text, double network data was fit to a model derived from the eight-chain model,[S5] which was adapted to account for the isotropic pre-swelling of the first network during double network synthesis. This model was derived as follows. First, following Arruda and Boyce,[S5] the initial, un-deformed state of the network is represented by 8 freely-jointed chains pointing from the center of a cube to its vertices. These chains initially have a root-mean-square end-to-end distance of $r_0 = \sqrt{n}l$, where n is the number of links of length l (equivalent to the number of Kuhn monomers) in each chain; the chain pointing to the vertex in the first octant has end-to-end vector

$$\vec{r} = r_0 \left(\frac{1}{\sqrt{3}}, \frac{1}{\sqrt{3}}, \frac{1}{\sqrt{3}} \right) \tag{6}$$

with similar expressions for the chains pointing to the other vertices of the cube. Upon isotropic pre-swelling from volume V_1 to volume V_2, all three dimensions stretch by $\lambda^* = \sqrt[3]{\frac{V_2}{V_1}}$, resulting in an end-to-end vector of

$$\vec{r} = r_0 \left(\frac{\lambda^*}{\sqrt{3}}, \frac{\lambda^*}{\sqrt{3}}, \frac{\lambda^*}{\sqrt{3}} \right) \tag{7}$$

When the network is then subjected to uniaxial extension along the x direction, the end-to-end vector at extension ratio λ is

$$\vec{r} = r_0 \left(\frac{\lambda^* \lambda}{\sqrt{3}}, \frac{\lambda^*}{\sqrt{3}\sqrt{\lambda}}, \frac{\lambda^*}{\sqrt{3}\sqrt{\lambda}} \right) \tag{8}$$

yielding an end-to-end distance of

$$r_{\lambda;\lambda^*} = r_0 \lambda^* \sqrt{\frac{\lambda^2}{3} + \frac{2}{3\lambda}} \tag{9}$$

Because all eight chains stretch by the same factors along each axis, the expression for the end-to-end distance is the same for the chains in the other octants.

We next assume that the number of chains per unit volume in the un-swollen network is N. After swelling, the number of chains per unit volume is $N\frac{V_1}{V2} = N/(\lambda^*)^3$; in tensile experiments conducted on double networks, this is the chain density in the "un-deformed" state of the double network. If the entropy of each chain with end-to-end distance r is $s(r)$, then the total work of deformation is

$$W = -T(S - S_0) = -T\frac{N}{(\lambda^*)^3}\left(s(r_{\lambda;\lambda^*}) - s(r_{1;\lambda^*})\right) \tag{10}$$

The stress at extension ratio λ is then

$$\sigma = \frac{dW}{d\lambda} = -T\frac{N}{(\lambda^*)^3}\frac{ds(r_{\lambda;\lambda^*})}{d\lambda} \tag{11}$$

For a Langevin chain with n links of length l,

$$\frac{ds}{dr} = -\frac{k}{l}\mathcal{L}^{-1}\left(\frac{r}{nl}\right) \tag{12}$$

where k is the Boltzmann constant and $\mathcal{L}^{-1}$ is the inverse Langevin function ($\mathcal{L}(\beta) = \coth(\beta) - \frac{1}{\beta}$). Following the chain rule, for a chain pre-stretched to λ^* and then subjected to uniaxial deformation to extension ratio λ,

$$\frac{ds(r_{\lambda;\lambda^*})}{d\lambda} = \frac{ds(r_{\lambda;\lambda^*})}{dr_{\lambda;\lambda^*}}\frac{dr_{\lambda;\lambda^*}}{d\lambda} \tag{13}$$

$$= \left[-\frac{k}{l}\mathcal{L}^{-1}\left(\frac{r_{\lambda;\lambda^*}}{nl}\right)\right]\left[r_0\lambda^*\frac{d\sqrt{\frac{\lambda^2}{3} + \frac{2}{3\lambda}}}{d\lambda}\right] \tag{14}$$

$$= \left[-\frac{k}{l}\mathcal{L}^{-1}\left(\frac{r_0\lambda^*\sqrt{\frac{\lambda^2}{3} + \frac{2}{3\lambda}}}{nl}\right)\right]\left[r_0\lambda^*\frac{\frac{2\lambda}{3} - \frac{2}{3\lambda^2}}{2\sqrt{\frac{\lambda^2}{3} + \frac{2}{3\lambda}}}\right] \tag{15}$$

Combining Eqns. 11 and 15 and substituting in $r_0 = \sqrt{n}l$ finally yields

$$\sigma = \frac{NkT}{3(\lambda^*)^2}\sqrt{n}\left[\mathcal{L}^{-1}\left(\frac{\lambda^*\sqrt{\frac{\lambda^2}{3}+\frac{2}{3\lambda}}}{\sqrt{n}}\right)\right]\left[\frac{\lambda-\frac{1}{\lambda^2}}{\sqrt{\frac{\lambda^2}{3}+\frac{2}{3\lambda}}}\right] \tag{16}$$

as given in the main text. Importantly, we note that this is *not* the same as replacing λ with $\lambda^*\lambda$ in the conventional expression for the 8-chain model; especially for networks with relatively short chains (low n) and/or high pre-swelling (large λ^*) it is necessary to include the isotropic pre-swelling in the functional form of the fit to obtain accurate values of n.

Representative fits to this model are shown in Figure S18. As seen in this figure, the model captures the low-strain behavior and the onset of strain stiffening with reasonable accuracy, comparable to empirical models used in prior work on networks with lower targeted M_x values.[S4,S6] At the highest strains (and particularly those above the inflection point in the high-strain response), the model predicts stronger strain-stiffening than is observed in the experimental samples; this deviation is likely due to the onset of bond-breakage in this regime.[S7]

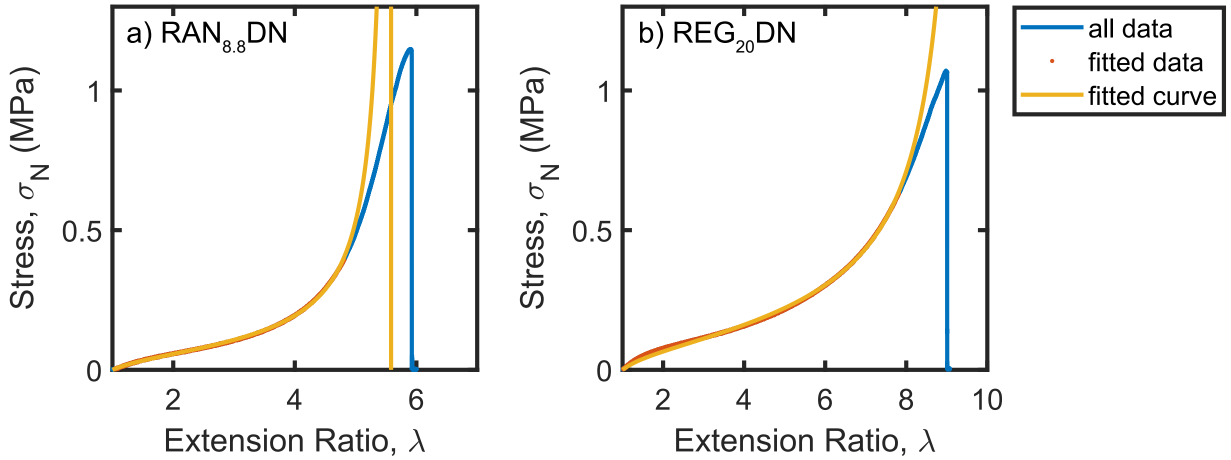

Figure S18: Representative fits to stress-strain data of (a) $RAN_{15}DN$ and (b)$REG20DN$.

As described in the main text, similar fits were carried out on all DN data. The fitted values of n and the equivalent strand lengths are summarized in the main text and Figure

S20, and the fitted values of NkT and the equivalent moduli are summarized in Figures S19 and S20. As shown in Figure S20, the strand lengths extracted from the model are approximately $\sim$ 5x higher than the targeted molecular weights between crosslinks. Part of this discrepancy arises from the fact that the networks were synthesized in solvent and deswollen prior to characterization; this deswelling leads to additional "stored length" which increases the apparent strand length by approximately a factor of 1.3 for the RAN networks and 1.8 for the REG networks (see below). The 8-chain model also assumes the networks are monodisperse and undergo affine deformation, neither of which is true in the experimental systems. Finally, as noted in the main text, the strain stiffening behavior of double network elastomers likely contains significant contributions from the evolution of the network structure (and particularly of the shortest percolating paths through the network) after bonds in the first network begin to break.[S8] As such, while the fitted chain lengths are expected to correctly capture the differences in the relative strain stiffening behavior of different double network elastomers, they may over-estimate the actual lengths of the individual chains in the network. The fitted NkT values over-estimate the experimental moduli by approximately a factor of 2, but are strongly correlated to the experimentally-measured moduli of the networks across both network types.

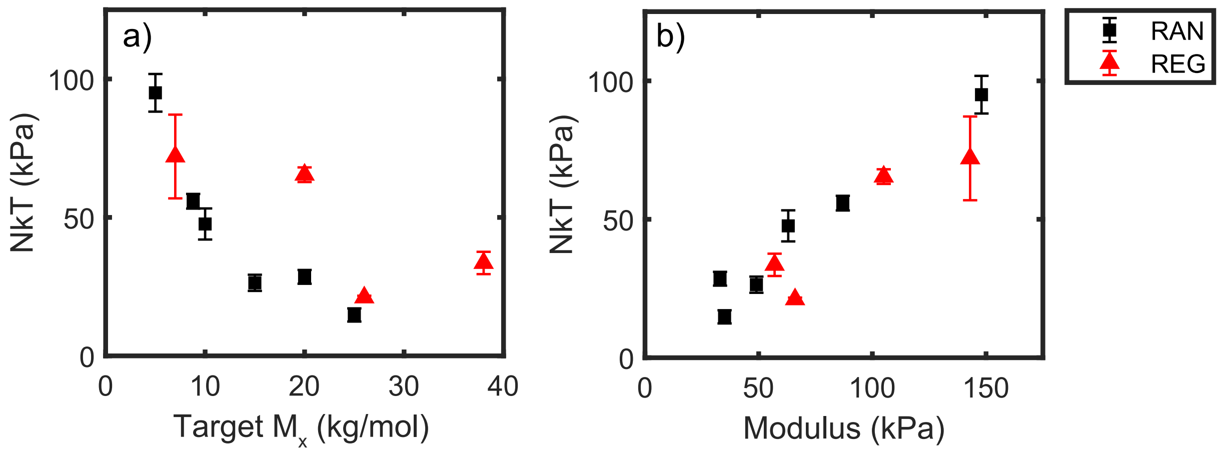

Figure S19: Fitted values of NkT as a function of (a) targeted M_x and (b) measured modulus for all double network samples. Error bars represent the standard deviation of fits to data from multiple sample replicates.

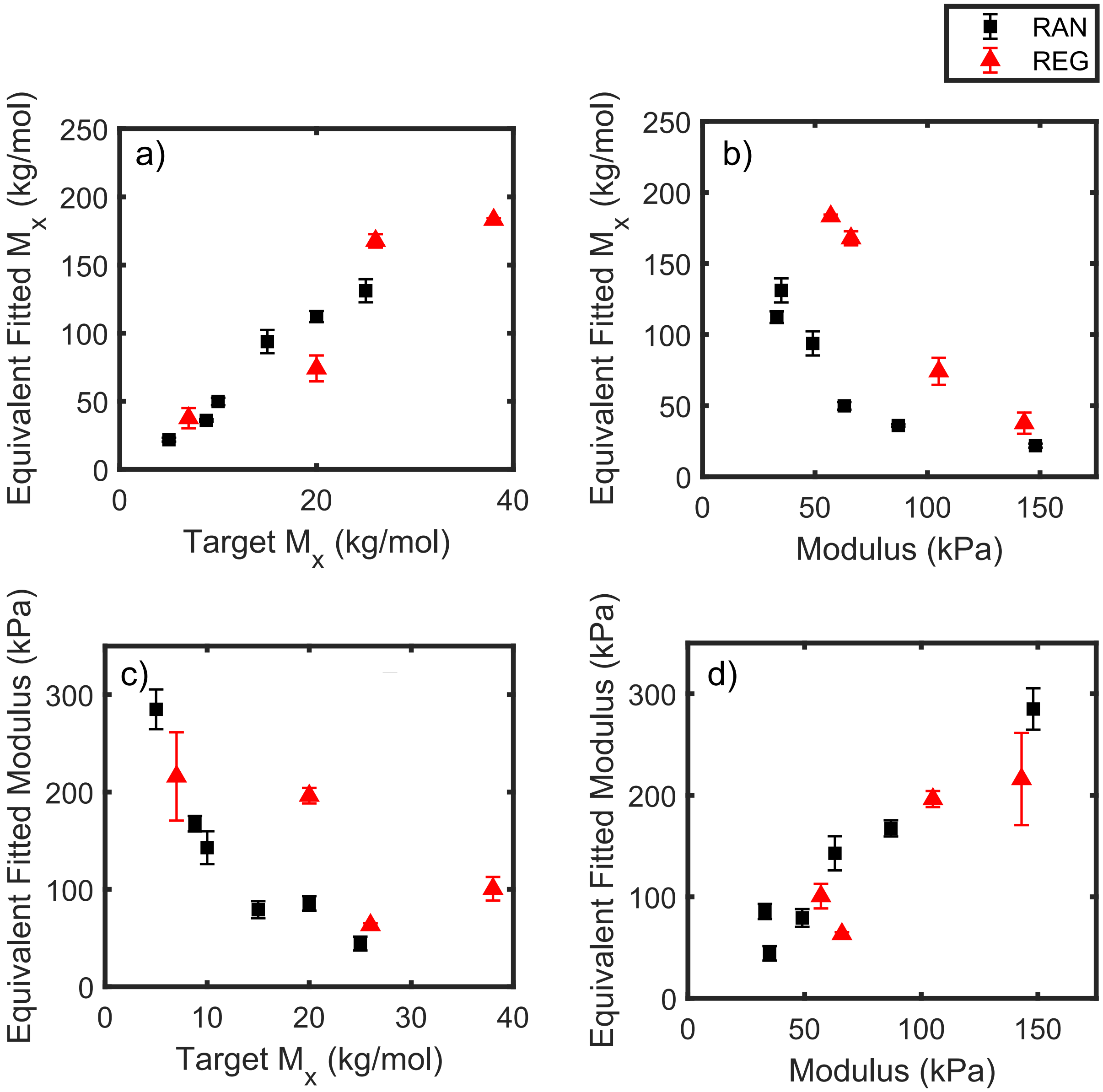

Figure S20: Data from Figure 5 in the main text and Figure S19, above, with the fitted number of Kuhn monomers (n) converted to equivalent molecular weight using a Kuhn monomer mass of 900 g/mol (derived from ref. S9) and the fitted value of NkT converted to the equivalent modulus using $E = 3NkT$.

Impact of Differences in Polymer Concentration During Network Synthesis

Due to the different solubilities of PnBA in the solvents used for the RAN and REG network syntheses (toluene and DMF, respectively), the parent RAN and REG networks were synthesized at slightly different polymer concentrations. RAN networks were synthesized from mixtures composed of 50 vol% monomer, corresponding to a volume fraction of polymer of 0.45 after polymerization. REG networks were synthesized from a mixture containing 20 wt% poly(n-butyl acrylate) star polymers in DMF, corresponding to a volume fraction of polymer of 0.18. The samples were then washed and dried prior to tensile measurements on the single networks and synthesis of the double networks. Here we summarize the key impacts that the differences in polymer concentration and deswelling prior to characterization have on the analysis and interpretation of the tensile data.

For a network synthesized at volume fraction of polymer ϕ_0, removing the solvent decreases the volume of the network to ϕ_0 of its original value; assuming deswelling is isotropic, all linear dimensions of the sample correspondingly decrease to $\phi_0^{1/3}$ of their original values (or a factor of 0.77 for RAN networks and 0.56 for REG networks synthesized under the conditions described above). If the polymer chains are assumed to be in their equilibrium conformations during synthesis, with end-to-end vector $\vec{r_0} = (x_0, y_0, z_0)$, then after deswelling/drying of the network, they have end-to-end vector $\vec{r_1} = \phi_0^{1/3}\vec{r_0} = \phi_0^{1/3}(x_0, y_0, z_0)$. Following uniaxial extension to extension ratio λ, the end-to-end vector becomes

$$\vec{r} = \phi_0^{1/3}\left(\lambda x_0, \frac{y_0}{\sqrt{\lambda}}, \frac{z_0}{\sqrt{\lambda}}\right) \tag{17}$$

The entropy change upon stretching from $\vec{r}_1$ to $\vec{r}$ is[S10]

$$\begin{aligned}\Delta S_{strand} &= -\frac{3k_B}{2r_0^2}(|\vec{r}|^2 - |\vec{r}_1|^2) \\ &= -\frac{3k_B}{2r_0^2}\phi_0^{2/3}\left(\left(\lambda^2 x_0^2 + \frac{y_0^2}{\lambda} + \frac{z_0^2}{\lambda}\right) - (x_0^2 + y_0^2 + z_0^2)\right)\end{aligned} \tag{18}$$

Following isotropic averaging ($x_0^2 = y_0^2 = z_0^2 = \frac{r_0^2}{3}$), the entropy change per strand becomes

$$\Delta S_{strand} = -\frac{k_B}{2}\phi_0^{2/3}\left(\lambda^2 + \frac{2}{\lambda} - 3\right) \tag{19}$$

Finally, for a network with a number-density of elastically effective strands of v (in the dried state characterized in the tensile experiment), the stress is

$$\sigma = kTv\phi_0^{2/3}\left(\lambda - \frac{1}{\lambda^2}\right) \tag{20}$$

and the modulus is

$$E = 3kTv\phi_0^{2/3} = 3kT\frac{\rho N_{av}}{M_x}\phi_0^{2/3} \tag{21}$$

as given in the main text.

The synthesis in solution and deswelling prior to characterization also affects the theoretical maximum extensibility of the networks. In the ideal chain limit, a chain of N Kuhn monomers of length b has a maximum end-to-end distance of

$$r_{max} = Nb \tag{22}$$

and an equilibrium root-mean-square end-to-end distance during network synthesis of

$$\langle r_0^2 \rangle^{1/2} = b\sqrt{N} \tag{23}$$

After deswelling, the RMS end-to-end distance is

$$\langle r^2 \rangle^{1/2} = b\sqrt{N}\phi_0^{1/3} \tag{24}$$

The extension ratio at which the chain is expected to reach its maximum extension is then

$$\lambda_{max} = \frac{r_{max}}{\langle r^2 \rangle^{1/2}} = \sqrt{N}\phi_0^{-1/3} \tag{25}$$

The "stored length" created by deswelling the networks thus increases the expected maximum extensibility of the RAN networks by a factor of approximately 1.3 and that of the REG networks by a factor of approximately 1.77.

Finally, we note that networks with the same modulus are expected to have the same maximum extensibility even when synthesized at different polymer concentrations. If each crosslink consists of N_x Kuhn monomers of mass M_0, $M_x = M_0 N_x$ and Eqn. 21 becomes

$$E = 3kT\frac{\rho N_{av}}{M_0 N_x}\phi_0^{2/3} \tag{26}$$

or

$$N_x = 3kT\frac{\rho N_{av}}{M_0 E}\phi_0^{2/3} \tag{27}$$

Combining Equations 25 and 27 yields

$$\begin{aligned} \lambda_{max} &= \sqrt{3kT\frac{\rho N_{av}}{M_0 E}\phi_0^{2/3}}\phi_0^{-1/3} \\ &= \sqrt{3kT\frac{\rho N_{av}}{M_0 E}} \end{aligned} \tag{28}$$

In the limit in which networks are well-described by a single average strand length, λ_{max} depends only on the modulus of the network and not on the polymer concentration at which it was synthesized. The differences in the onsets of strain stiffening between RAN and REG networks shown in Figs. 4d and 5b in the main text are thus not attributable to the

different polymer fractions at which the parent networks were synthesized, and must instead arise from differences in their topologies and/or distributions of strand lengths.

References

(S1) Test Method for Tensile Properties of Plastics. https://doi.org/10.1520/d0638-22.

(S2) Jullian, N.; Leonardi, F.; Grassl, B.; Peyrelasse, J.; Derail, C. Rheological characterization and molecular modeling of poly(n-butyl acrylate). *Applied Rheology* **2010**, *20*, 33685, DOI: 10.3933/APPLRHEOL-20-33685.

(S3) Rubinstein, M.; Panyukov, S. Nonaffine Deformation and Elasticity of Polymer Networks. *Macromolecules* **1997**, *30*, 8036–8044, DOI: 10.1021/ma970364k.

(S4) Millereau, P.; Ducrot, E.; Clough, J. M.; Wiseman, M. E.; Brown, H. R.; Sijbesma, R. P.; Creton, C. Mechanics of elastomeric molecular composites. *Proceedings of the National Academy of Sciences* **2018**, *115*, 9110–9115, DOI: 10.1073/pnas.1807750115.

(S5) Arruda, E. M.; Boyce, M. C. A three-dimensional constitutive model for the large stretch behavior of rubber elastic materials. *Journal of the Mechanics and Physics of Solids* **1993**, *41*, 389–412, DOI: 10.1016/0022-5096(93)90013-6.

(S6) Ducrot, E.; Creton, C. Characterizing Large Strain Elasticity of Brittle Elastomeric Networks by Embedding Them in a Soft Extensible Matrix. *Advanced Functional Materials* **2016**, *26*, 2482–2492, DOI: 10.1002/adfm.201504536.

(S7) Ducrot, E.; Chen, Y.; Bulters, M.; Sijbesma, R. P.; Creton, C. Toughening Elastomers with Sacrificial Bonds and Watching Them Break. *Science* **2014**, *344*, 186–189, DOI: 10.1126/science.1248494.

(S8) Yin, Y.; Bertin, N.; Wang, Y.; Bao, Z.; Cai, W. Topological origin of strain induced damage of multi-network elastomers by bond breaking. *Extreme Mechanics Letters* **2020**, *40*, 100883, DOI: 10.1016/j.eml.2020.100883.

(S9) Ahmad, N. M.; Lovell, P. A.; Underwood, S. M. Viscoelastic properties of branched polyacrylate melts. *Polymer International* **2001**, *50*, 625–634, DOI: 10.1002/pi.672.

(S10) Hiemenz, P.; Lodge, T. *Polymer Chemistry, Second Edition*; Taylor & Francis, 2007.
